# Comparative Efficacy of Chinese Herbal Injections for Pulmonary Heart Disease: A Bayesian Network Meta-Analysis of Randomized Controlled Trials

**DOI:** 10.3389/fphar.2020.00634

**Published:** 2020-05-07

**Authors:** Kaihuan Wang, Jiarui Wu, Haojia Wang, Xiaojiao Duan, Dan Zhang, Yingzi Wang, Mengwei Ni, Shuyu Liu, Ziqi Meng, Xiantao Zeng, Xiaomeng Zhang

**Affiliations:** ^1^Department of Clinical Chinese Pharmacy, School of Chinese Materia Medica, Beijing University of Chinese Medicine, Beijing, China; ^2^Center for Evidence-Based and Translational Medicine, Zhongnan Hospital of Wuhan University, Wuhan, China

**Keywords:** network meta-analysis, Bayesian model, pulmonary heart disease, Chinese herbal injection, Shenfu injection, Shenmai injection, Shenqi Fuzheng injection

## Abstract

**Background:**

Given the severity of pulmonary heart disease and the wide utilization of Chinese herbal injections, this network meta-analysis was devised to assess the comparative efficacy of seven Chinese herbal injections (Ciwujia injection, Dazhuhongjingtan injection, Huangqi injection, Shenfu injection, Shengmai injection, Shenmai injection, and Shenqi Fuzheng injection) that were combined with Western medicines in the treatment of pulmonary heart disease.

**Methods:**

A literature search was performed in PubMed, Cochrane Library, EMBASE, Chinese Biological Medicine Database, China National Knowledge Infrastructure, Wanfang Database, and the Chinese Scientific Journal Database from their inception to July14, 2019. This network meta-analysis was conducted in accordance with *a priori* eligibility criteria and methodological quality recommendations. Data analysis was performed with WinBUGS 1.4.3 and Stata 13.0 software focusing on clinical effectiveness rate, arterial blood gas analysis, hemorheology and hemodynamic indexes and right ventricular dimensions. In addition to the odds ratio or mean difference in various outcomes, the ranking probability of interventions calculated by the surface under the cumulative ranking area curve was demonstrated. The surface under the cumulative ranking area was equal to the rank of the intervention and was aimed to assess the best intervention.

**Results:**

Ultimately, 118 randomized controlled trials including 10,085 patients were included. Integrating the outcome results, all eligible Chinese herbal injections plus Western medicines were superior to Western medicines alone, especially Shenfu injection+ Western medicines, Shenmai injection+ Western medicines, and Shenqi Fuzheng injection+ Western medicines. Regarding safety, the drip rate was an essential element for clinicians to consider during treatment.

**Conclusions:**

In conclusion, Shenfu injection+ Western medicines, Shenmai injection+ Western medicines and Shenqi Fuzheng injection+ Western medicines may be potential optimal treatments for pulmonary heart disease. A larger sample size and high-quality randomized controlled trials are needed to confirm and support this network meta-analysis.

## Introduction

Pulmonary heart disease (PHD), a pathologic condition that increases pulmonary vascular resistance and pulmonary artery pressure, is caused by lesions in bronchial and lung tissue and the pulmonary vascular system and leads to the irreversible development of pulmonary hypertension and ultimate overload of the right heart or even right heart failure (Forfia et al., 2013; Wang et al., 2016).With an estimated average prevalence of 0.46% worldwide for PHD, the heavy burden of these patients and their families has also been placed on society (Yun et al., 2016). Moreover, the estimated mortality rate among hospitalized patients is between 12.5% and 14.5% globally (Ye and Lu, 2004; He and Liu, 2014). At present, the predominant treatment for PHD is Western medicine (WM), including oxygen therapy, antibiotics, diuretics, vasodilators, antiarrhythmic agents, and others. However, the efficacy of these treatments is unsatisfactory (Chen et al., 2013; Shi et al., 2015).

The use of traditional Chinese medicine combined with WM has been extensively promoted in routine practice in China. In light of traditional Chinese medicine theories, PHD is an aspect of “lung distension” and “dyspnea” and is caused by the lungs and heart. The clinical principle mainly emphasizes strengthening bodily resistance to eliminate pathogenic factors (Yu, 2009; Ruan et al., 2018). Chinese herbal injections are an indispensable part of traditional Chinese medicine and play a vital role in treating PHD. For the past twenty years, their effectiveness has been confirmed in clinical trials (Liao et al., 2004; China association of traditional Chinese medicine of lung diseases, 2014; Yun et al., 2016).

However, no clinical trials have focused on the comparative efficacy of administering various Chinese herbal injections simultaneously, which may cause difficulties for clinicians when choosing an optimal regimen. Network meta-analysis (NMA) can help fill this void, as NMA is an extension of conventional pairwise meta-analysis and can synthesize the available evidence to enable a simultaneous comparison and assessment of the best intervention amongst those that lack head-to-head evaluations (Lu and Ades, 2004; Singh et al., 2016; Cipriani et al., 2018; Cai et al., 2018). In this context, this study incorporated seven Chinese herbal injections, namely, Ciwujia, Dazhuhongjingtan, Huangqi, Shenfu, Shengmai, Shenmai, and Shenqi Fuzheng injections, to comprehensively evaluate the efficacy of these injections combined with WM using NMA. The goal of this NMA was to inform clinical practice and provide additional insights for the selection of PHD treatments.

## Methods

This NMA was performed in accordance with The PRISMA Extension Statement for Reporting of Systematic Reviews Incorporating Network Meta-analyses of Health Care Interventions (Hutton et al., 2015). A completed PRISMA checklist is included as an additional file (**Presentation File**).

### Search Strategy

In this NMA, a comprehensive data search was conducted using the following electronic databases from their inception to July14, 2019: PubMed, Cochrane Library, EMBASE, Chinese Biological Medicine Database, China National Knowledge Infrastructure, Wanfang Database, and the Chinese Scientific Journal Database. The method of combining MeSH terms with free text search terms was applied to the search. Using PubMed as an example, two reviewers developed the search strategy as follows:(randomized controlled trial[Publication Type] OR controlled clinical trial[Publication Type] OR random*[All fields]) AND (ciwujia[Title/Abstract]OR acanthopanax[Title/Abstract] OR dazhuhongjintian[Title/Abstract] OR rhodiola[Title/Abstract]OR huangqi[Title/Abstract] OR astragalus[Title/Abstract] OR shenfu[Title/Abstract] OR shengmai[Title/Abstract] OR shenmai[Title/Abstract] OR shenqi fuzheng[Title/Abstract] OR yiqifumai[Title/Abstract]) AND (pulmonary heart disease[MeSH terms] OR pulmonary heart disease*[Title/Abstract] OR corpulmonale[Title/Abstract]) (See the **Presentation File** for more details about the search strategy).

### Inclusion Criteria

#### Types of Studies

Randomized controlled trials (RCTs) that reported the efficacy of the seven Chinese herbal injections combined with WM for treating PHD were eligible. No limitation on language, publication year, or publication status was applied. If a study was published more than once, we included only the first publication.

#### Types of Participants

Patients who suffered from PHD and were diagnosed according to the specific diagnostic criteria were included. Gender, ration and nationality were unrestricted. Patients would be excluded if they had severe complications.

#### Types of Interventions

Eligible RCTs were not limited to two-arm RCTs. All RCTs included WM, including treatments to control respiratory tract infection and improve respiratory and heart failure, as well as anti-arrhythmic drugs. The experimental group was administered one of the eligible Chinese herbal injections and WM, while the control group was administered the same WM alone or in combination with another Chinese herbal injection. If patients had complications during the therapeutic process, the appropriate therapy needed to be adopted. No restriction was placed on dosage or duration, but for a study to be eligible, it needed to include the specific dosage of the Chinese herbal injection.

#### Types of Outcomes

(1) Clinical effectiveness rate. The clinical effectiveness rate was calculated with the following formula: (number of remarkable recovery patients + number of basic recovery patients)/total number of patients * 100%. To be considered a remarkable recovery, patients needed to show complete amelioration of clinical symptoms and improvement of cardiac function by two levels. For a basic recovery, patients needed to show relief from clinical symptoms and an improvement in cardiac function by one level. Unaltered or worsened clinical symptoms and cardiac function were regarded as deterioration. Cardiac function classification conformed to the standard issued by the New York Heart Association in the United States. (2) Arterial blood gas analysis (partial pressure of arterial oxygen, partial pressure of arterial carbon dioxide). (3) Hemorheology (the level of whole blood viscosity and the level of fibrinogen). (4) Hemodynamics (mean pulmonary arterial pressure). (5) Right ventricular dimension. RCTs were eligible if they reported one of the aforementioned outcomes. The safety of the intervention (adverse drug reactions/adverse drug events (ADRs/ADEs)) was also summarized.

### Data Extraction and Quality Assessment

The initial literature screening process was conducted by two reviewers through reading titles and abstracts. Then, the full-text versions of potential articles were obtained for further assessment. Any discrepancies between the two reviewers were resolved by discussion or consultation with a third reviewer. Next, data were extracted in accordance with the predesigned form, including the first author name, publication year, patient characteristics (sample size, gender, age, patients' baseline, and disease duration), intervention details, duration, outcomes, study design and the domains of risk of bias.

The Cochrane Collaboration risk of bias tool was used to evaluate the quality of the eligible RCTs. The following items were accessed: sequence generation (selection bias), allocation concealment (selection bias), blinding of patients and personnel (performance bias), blinding of outcomes assessors (detection bias), incomplete outcome data (attrition bias), selective reporting (reporting bias), and other biases. Each bias had three levels: “low risk”, “unclear risk” and “high risk”. Quality assessment was performed by two reviewers, and any conflicts during this process were solved by discussion or consultation.

### Data Analysis

For each outcome, we carried out a Bayesian NMA to compare efficacy between eligible Chinese herbal injections. The calculation was performed by WinBUGS 1.4.3 software, and correlative graphical representation was depicted using Stata 13.0 software. The OR ratios (OR) and mean differences (MD) with their 95% confidence intervals (95% CI) were produced in a random-effects model for binary and continuous outcomes, respectively. When the OR included 1.00 or the MD included 0.00, the meta-analysis result was deemed not statistically significant. In the analysis process, the number of iterations was set to 200,000, and the first 10,000 were used for the annealing algorithm to eliminate the impact of the initial value.

Furthermore, a surface under the cumulative ranking area (SUCRA) curve was used to estimate the ranking probabilities for each intervention, which ranged from 0 to 100%.Interventions with larger SUCRA values were considered better interventions (Chang and Guo, 2017; Cai et al., 2018). To determine the most efficacious injection for the PHD treatment, a cluster analysis for different outcomes was carried out (Tang et al., 2016). A funnel plot was also depicted to estimate the publication bias of outcomes included in more than10 RCTs. The network graph is displayed as well.

This NMA was based on previous publications, and thus, ethical approval was unnecessary.

## Results

### Search Results

A total of 2,421 records were identified from the seven databases. Then, 1,159 records were removed for duplication, and 258 records were excluded though scanning titles and abstracts because they were reviews, animal experiments or irrelevant studies. The full texts of the remaining records were screened, and 886 records were excluded for the following reasons: (1) It was not an RCT, or it was an RCT with inappropriate randomization (n = 28). (2) It was a retrospective study (n = 3). (3) The intervention did not meet the inclusion criteria (n = 620). (4) The RCT did not mention a standard diagnostic criterion (n = 128). (5) The outcomes did not meet the inclusion criteria (n = 46). (6) The report contained duplicate data (n = 48). (7) It was an RCT without an available full-text report (n = 13). Ultimately, 118 RCTs were included in this NMA (**Presentation File**). They were all conducted in China and published from 1996 to 2017.

### Inclusion Studies and Characteristics

**Figure 1:**
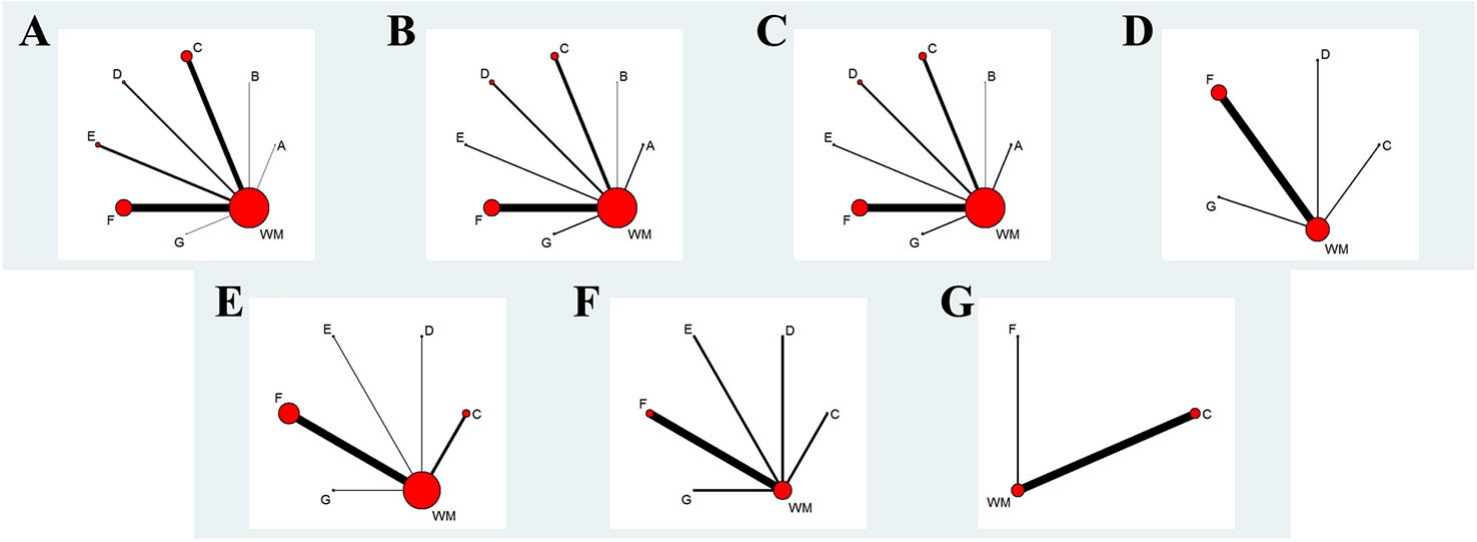
Network graph of different outcomes. **(A)** The Clinical Effectiveness Rate; **(B)** Partial Pressure of Arterial Oxygen; **(C)** Partial Pressure of Arterial Oxygen Carbon Dioxide; **(D)** The Level of Whole Blood Viscosity; **(E)** The Level of Fibrinogen; **(F)** Mean Pulmonary Arterial Pressure; **(G)** Right Ventricular Dimension. A: Ciwujia injection+WM; B: Dazhuhongjingtian injection+WM; C: Huangqi injection+WM; D: Shenfu injection+WM; E: Shengmai injection+WM; F: Shenmai injection+WM; G: Shenqi Fuzheng injection+WM.

One hundred and eighteen two-arm RCTs containing 10,085 patients (5,241 patients in the experimental group, 4,844 patients in the control group) were eligible for this NMA. Among the patients, the majority were middle-aged and elderly people, and 6,408 (63.5%) of the 10,085 patients were men. A total of eight interventions were evaluated: Ciwujia injection+WM, Dazhuhongjingtian injection+WM, Huangqi injection+WM, Shenfu injection+WM, Shenmai injection+WM, Shenmai injection+WM, Shenqi Fuzheng injection+WM and WM (Detailed information of included Chinese herbal injection were showed in the **Table 1**). WM involving therapy for controlling respiratory tract infections, improving respiratory and heart failure, and regulating electrolyte and acid-base balance were adopted in the control groups of all eligible RCTs. The interventions of the experimental group were as follows: on the basis of their corresponding control group, three RCTs administered Ciwujia injections, one RCT administered Dazhuhongjingtian injections, 31 RCTs administered Huangqi injections, 11 RCTs administered Shenfu injections, 15 RCTs administered Shengmai injections, 54 RCTs administered Shenmai injections, and three RCTs administered Shenqi Fuzheng injections. Among the eligible RCTs, 23 RCTs exceed the specification dosage when administering a Chinese herbal injection (Huangqi injection, 20 RCTs; Shenfu injection, two RCTs; Shengmai injection, one RCT). In addition, 26 eligible RCTs did not follow the specification for utilizing menstruum (Dazhuhongjingtian injection, one RCT; Shenfu injection, four RCTs; Shengmai injection, four RCTs; Shenmai injection, 17 RCTs). Fifteen RCTs were prescribed a Chinese herbal injection based on syndrome differentiation. The eligible Chinese herbal injections were given *via* intravenous drip once a day, except for two RCTs, which reported an administration twice a day, and one RCT that did not mention it. The RCT duration ranged from 7 to 42 days. In terms of outcomes, 83.1% of the RCTs reported a clinical effectiveness rate, 25.4% of the RCTs mentioned arterial blood gas analysis, 15.3% of the RCTs evaluated hemorheology results, 5.9% of the RCTs tested the hemodynamic dimension and 4.2% of the RCTs measured the right ventricular dimension. **Table 2** summarizes the characteristics of the eligible RCTs, and **Figure 1** illustrates the network graphs of the various eligible outcome comparisons.

**Table 1:** Detailed information on Chinese herbal injections.

Chinese herbal injection (Name of the formulation)	Name of the herbal drug	Scientific name of the plant	Composition with Chinese pinyin
Ciwujia injection	ACANTHOPANACIS SENTICOSI RADIX ET RHIZOMA SEU CAULIS	*Acanthopanax senticosus* (Rupr.et Maxim.) Harms	Ciwujia
Dazhuhongjingtian injection	RHODIOLAE CRENULATAE RADIX ET RHIZOMA	*Rhodiola crenulata* (Hook. f. et Thoms.) H. Ohba	Dazhuhongjingtian
Huangqi injection	ASTRAGALI RADIX	*Astragalus membranaceus* (Fisch.) Bge.var. *mongholicus* (Bge.) Hsiao *or Astragalus membranaceus* (Fisch.) Bge.	Huangqi
Shenfu injection	GINSENG RADIX ET RHIZOMA RUBRA, ACONITI LATERALIS RADIX PRAEPARATA	*Panaxginseng* C.A.Mey., *Aconitum carmichaeli* Debx.	Hongshen, Fuzi
Shengmai injection	GINSENG RADIX ET RHIZOMA RUBRA, OPHIOPOGONIS RADIX, SCHISANDRAE CHINENSIS FRUCTUS	*Panaxginseng* C.A.Mey.*, Ophiopogon japonicas* (L.f) Ker-GawL*, Schisandra chinensis* (Turcz.) Baill.	Hongshen, Maidong, Wuweizi
Shenmai injection	GINSENG RADIX ET RHIZOMA RUBRA, OPHIOPOGONIS RADIX	*Panaxginseng* C.A.Mey.*, Ophiopogon japonicas* (L.f) Ker-GawL,	Hongshen, Maidong
Shenqi Fuzheng injection	ASTRAGALI RADIX, CODONOPSIS RADIX	*Astragalus membranaceus* (Fisch.) Bge.var. *mongholicus (Bge.) Hsiao or Astragalus membranaceus* (Fisch.) Bge.*, Codonopsis pilosula* (Franch.) Nannf. *or Codonopsis pilosula* Nannf. var. *modesta* (Nannf.) L. T. Shen *or Codonopsis tangshen* Oliv.	Huangqi, Dangshen

**Table 2:** Characteristics of the included randomized controlled trials.

Study ID	Sample Size (E/C)	Gender (M/F)	Age (Year)	Disease Duration (Year)	Experimental Group	Chinese Herbal Injection Solution Content	Control Group	Course of Treatment (Days)	Outcomes
Qiu YL 1996	27/27	39/15	E:46.7 C:49.5	E:6–12 C:5–13	CWJI40-60 ml+WM, ivgtt, qd	5%GS500 ml	WM	15 d	1
Zhu YH 1999	32/32	37/27	E:59.0 ± 2.5 C:59.0 ± 3.0	E:11.0 ± 2.5 C:11.0 ± 2.6	CWJI40 ml+WM, ivgtt, qd	5%GS250 ml	WM	10 d	1,2
Li DS 2003	30/30	33/27	E:67.1 ± 6.2 C:66.3 ± 5.6	NR	CWJI40 ml+WM, ivgtt, qd	NR	WM	14 d	1,2
Lin HQ 2014	54/54	68/40	E:66.7 ± 7.1 C:69.2 ± 5.9	NR	DZHJTI10 ml+WM, ivgtt, qd	5%GS/0.9%NS 250 ml	WM	10 d	1,2
Yang ZY 1997	65/65	107/23	E:57.23 C:56.50	NR	HQI20 ml+WM, ivgtt, qd	5%GS300 ml	WM	15 d	1
Sun Q 1998	42/30	44/28	E:61.5 C:63.2	NR	HQI20 ml+WM, ivgtt, qd	5%GS250 ml	WM	12 d	1,3,6
Wang B 1999*	51/38	53/36	E:58.3 ± 11 C:60.2 ± 13	E:17 C:16	HQI40-60 ml+WM, ivgtt, qd	0.9%NS	WM	14 d	1
Gao DF 1999	41/38	45/34	E:65 ± 8 C:63 ± 10	NR	HQI20 ml+WM, ivgtt, qd	5%GS250 ml	WM	28 d	1
Zhou B 1999*	64/59	76/47	E:59 ± 15 C:57 ± 15	22 ± 16	HQI30 ml+WM, ivgtt, qd	10%GS250 ml	WM	10 d	5,6
Bai R 1999	25/25	26/24	E:62.92 ± 10.15 C:61.72 ± 9.88	NR	HQI10-20 ml+WM, ivgtt, qd	10%GS100 ml	WM	10–14 d	1,6
Zhao LY 2000	16/13	24/5	E:50–75 C:52–77	NR	HQI30 ml+WM, ivgtt, qd	5%GS200 ml	WM	10 d	1,6
Zhang LJ 2000	70/70	82/58	E:62.5 C:61.7	NR	HQI20 ml+WM, ivgtt, qd	5%GS250 ml	WM	28–42 d	1,6
Wang KX 2000*	40/38	49/29	E:68.5 C:70.5	NR	HQI80 ml+WM, ivgtt, qd	5%GS200 ml	WM	7–14 d	1,6
Zhu ZY 2001*	61/62	80/43	E:61 ± 15 C:60 ± 16	E:7–32 C:6–33	HQI30 ml+WM, ivgtt, qd	5%GS250 ml	WM	10 d	1,2,6
Ma ZP 2001*	43/43	67/19	E:53–78 C:51–83	5–40	HQI50 ml+WM, ivgtt, qd	5%GS200 ml	WM	10 d	1
Zhu H 2001	30/30	35/25	E:67.5 C:67.5	E:8.9 C:8.8	HQI40 ml+WM, ivgtt, qd	5%GS250 ml	WM	7 d	1,3,6
Zhang DJ 2002*	42/42	57/27	E:50.1 C:49.3	E:8.5 C:8.2	HQI40 ml+WM, ivgtt, qd	5%GS200 ml	WM	14 d	1,6
Yin J 2002*	50/45	55/40	E:50–75 C:47–73	6-35	HQI20 ml+WM, ivgtt, qd	5%GS250 ml	WM	15 d	1,5,6
Sun GY 2002	40/40	47/33	E:71.5 C:70.5	NR	HQI50 ml+WM, ivgtt, qd	5%GS250 ml	WM	10 d	1
Zou Q 2002	40/32	58/14	E:65 C:63	E:7.3 C:7.5	HQI40 ml+WM, ivgtt, qd	5%GS250 ml	WM	15 d	1
Fan XY 2003*	46/46	70/22	E:63.6 ± 6.8 C:64.5 ± 7.2	NR	HQI40 ml+WM, ivgtt, qd	5%GS	WM	10–15 d	2
Zhou LJ 2003	60/60	81/39	E:68.5 C:65.8	E:5–45 C:6–48	HQI40 ml+WM, ivgtt, qd	5%GS250 ml	WM	10–14 d	1,2,6
Cai J 2003*	56/56	79/33	E:51–82 C:46–79	8–36	HQI40 ml+WM, ivgtt, qd	5%GS250 ml	WM	14 d	1,6
Wang W 2004	60/60	77/43	E:65 ± 5.3 C:64.5 ± 6.8	E:7–21 C:6–19	HQI1 g/(kg·d)+WM, ivgtt	5%GS350 ml	WM	15 d	1
Li YH 2004	36/35	46/25	E:61.54 C:62.35	E:16 C:15	HQI30 ml+WM, ivgtt, qd	5%GS250 ml	WM	14 d	1,2,3,6
Wu LW 2003	30/30	39/21	E:62 ± 10.5 C:60 ± 11.5	E:15.0 ± 6.5 C:14.5 ± 6.0	HQI30 ml+WM, ivgtt, qd	5%GS	WM	10 d	1,6
Zhu YH 2005	32/28	36/24	E:72.02 ± 6.21 C:71.42 ± 7.11	NR	HQI20 ml+WM, ivgtt, qd	GS/0.9%NS250-500 ml	WM	14 d	1,2,4,5
Ma L 2005*	68/68	84/52	E:60–76 C:58–78	NR	HQI50 ml+WM, ivgtt, qd	NR	WM	7 d	1
Guo HD 2005	40/40	60/20	E:60.3 C:59.3	NR	HQI20 ml+WM, ivgtt, qd	5%GS200 ml	WM	7–10 d	1,6
Wang L 2007	98/98	114/82	E:56 C:57.6	E:3–12 C:5–14	HQI40-60 ml+WM, ivgtt, qd	5%GS/0.9%NS 500 ml	WM	NR	1
Hui SL 2008	50/50	77/23	E:58.6 C:58.2	NR	HQI30 ml+WM, ivgtt, qd	5%GS250 ml	WM	10–14 d	1,6
Chen Xm 2009	40/38	61/17	E: < 60 15 cases, 60–70 10 cases, > 70 15 cases C: < 60 14 cases, 60–70 9 cases, > 70 15 cases	E: < 0.5 11 cases, 0.5–1 1 9 cases, > 110 cases C: < 0.5 10 cases, 0.5–1 20 cases, > 1 8 cases	HQI30-40 ml+WM, ivgtt, qd	5%GS250 ml	WM	10 d	2,5,6
Xiao W 2012	160/160	200/120	E:66.9 C:66.8	E: < 10 93 cases, > 10 67 cases C: < 10 88 cases, ≥10 72 cases	HQI40 ml+WM, ivgtt, qd	5%GS250 ml	WM	14 d	1
Liu MS 2012	15/15	15/15	E:52.6 C:52.2	E:3.8 C:3.8	HQI30 ml+WM, ivgtt, qd	5%GS200 ml	WM	10 d	1
Li YQ 2014*	35/35	38/32	E:54.2 ± 2.4 C:55.2 ± 2.3	NR	HQI20 ml+WM, ivgtt, qd	5%GS	WM	14 d	1
Kong XM 2003	20/20	26/14	62.9	NR	SFI20 ml+WM, ivgtt, qd	5% GS250 mL	WM	14 d	2
Li XM 2006	30/30	35/25	71.4	8–13	SFI1 mg/kg+WM, ivgtt, qd	5% GS250 mL	WM	10 d	1,4
Li LZ 2007*	48/48	58/38	E:62.9 C:63.2	E:15.4 C:14.8	SFI40 ml+WM, ivgtt, qd	5% GS250 mL	WM	15 d	1,2
Fan DB 2009	62/62	69/55	E:62.42 C:61.89	E:16.56 C:16.24	SFI40 ml+WM, ivgtt, qd	5% GS250 mL	WM	14 d	1
Shen XX 2011	40/42	58/24	E:68.32 ± 8.12 C:64.54 ± 8.13	NR	SFI1 mg/kg+WM, ivgtt, qd	5% GS100 mL	WM	10 d	1,2
Wu DEJ 2011	32/32	50/14	E:69.10 ± 7.80 C:68.90 ± 7.40	NR	SFI30 ml+WM, ivgtt, qd	5% GS100 mL	WM	14 d	1
Ru HG 2011*	24/24	35/13	E:62.6 ± 7.8 C:63.6 ± 8.1	NR	SFI60 ml+WM, ivgtt, qd	5% GS150 mL	WM	10 d	1
Guo FC 2014	45/45	58/32	E:72.2 ± 8.1 C:70.4 ± 7.8	NR	SFI50 ml+WM, ivgtt, bid	5% GS	WM	10 d	1,2
Lu Q 2014	78/64	90/52	E:61.4 C:60.7	NR	SFI40 ml+WM, ivgtt, qd	5% GS250 mL	WM	14 d	3
Qian XL 2015	33/33	39/27	E:71.2 ± 3.4 C:68.5 ± 2.9	NR	SFI50 ml+WM, ivgtt, bid	5% GS150-250 mL	WM	14 d	1
Lin B 2017	30/30	50/10	E:67.1 ± 2.3 C:68.4 ± 1.8	NR	SFI40 ml+WM, ivgtt, qd	5% GS250 mL	WM	14 d	1
Chen DZ 2000	40/40	46/34	E:62 C:63	17	SI20 ml+WM, ivgtt, qd	5%GS100 ml	WM	15 d	1
Gao YD 2001	58/54	81/31	66.8	NR	SI25 ml+WM, ivgtt, qd	5%GS250 ml	WM	10 d	1
Chen XZ 2002	124/102	160/66	75.6	NR	SI20 ml+WM, ivgtt, qd	5%GS250–500 ml	WM	15 d	1
Li DH 2003	77/77	82/72	E:63.5 C:63	NR	SI30 ml+WM, ivgtt, qd	5%GS250 ml	WM	14 d	1
Liu ZQ 2994	30/30	33/27	E:56 C:55	E:9 C:8	SI80-100 ml+WM, ivgtt, qd	5%GS500 ml	WM	10 d	2,4
Li JH 2004	31/31	42/20	E:71.87 ± 4.78 C:70.87 ± 6/97	E:18.38 ± 1.05 C:19.47 ± 6.64	SI60 ml+WM, ivgtt, qd	5%GS500 ml	WM	21 d	1
Yu JX 2005	34/30	48/16	E:67.5 C:66.8	NR	SI40-60 ml+WM, ivgtt, qd	5%GS250 ml	WM	14 d	1
Ma DT 2006	69/62	95/36	67	5-19	SI60 ml+WM, ivgtt, qd	5%GS100–150 ml	WM	7-10 d	1,6
Dong XF 2007	87/89	95/81	E:67 C:65	E:19 C:18	SI40 ml+WM, ivgtt, qd	5%GS250 ml	WM	7 d	1
Chen GY 2008	40/40	66/14	E:66 C:64	E:8–25 C:9–22	SI50 ml+WM, ivgtt, qd	5%GS250 ml	WM	14 d	1,3
Wang X 2008	35/35	51/19	E:63 C:63	E:13 C:12	SI40 ml+WM, ivgtt, qd	5%GS250 ml	WM	14 d	2
Sun T 2009	75/63	69/69	E:53.8 C:652.6	NR	SI40 ml+WM, ivgtt, qd	5%GS/0.9%NS250 ml	WM	14 d	1
Li YQ 2009	26/25	30/21	E:69.25 ± 3.25 C:59.32 ± 5.17	E:15.18 ± 1.03 C:16.57 ± 5.76	SI40-60 ml+WM, ivgtt, qd	5%GS350–500 ml	WM	21 d	1
Chen ZJ 2010	50/50	47/53	E:66.8 C:67.1	NR	SI60 ml+WM, ivgtt, qd	5%GS200 ml	WM	14 d	1,6
Liu HL 2010	60/60	91/29	E:67 ± 10 C:66 ± 9	E:13 ± 4 C:12 ± 5	SI50 ml+WM, ivgtt, qd	5%GS250 ml	WM	14 d	1,6
Jiang QF 1996	45/30	61/14	55.7	NR	SMI30 ml+WM, ivgtt, qd	5%GS250 ml	WM	10 d	2
Guo XH 1999	36/22	43/15	E:64.2 C:64.7	NR	SMI30 ml+WM, ivgtt, qd	5%GS250 ml	WM	10 d	1
Ye PX 1999	50/40	72/18	E: < 50 6cases, 50–70 38cases > 70 6 cases C: < 50 5 cases, 50–70 31 cases, > 70 4 cases	NR	SMI60 ml+WM, ivgtt, qd	10%GS250 ml	WM	10 d	1
Cai ZW 1999	37/36	37/36	E:69 C:68	3–20	SMI30 ml+WM, ivgtt, qd	5%GS250 ml	WM	10 d	1
Ye px 1999	32/31	45/18	64	NR	SMI60 ml+WM, ivgtt, qd	10%GS250 ml	WM	10 d	3
Song ZB 1999	37/37	66/8	E:72.93 ± 7.59 C:71.68 ± 6.72	E:16.78 ± 8.14 C:15.93 ± 7.84	SMI30 ml+WM, ivgtt, qd	5%GS250 ml	WM	7 d	1
Zhu XF 2001	53/47	72/28	E:65 C:63	NR	SMI30 ml+WM, ivgtt, qd	5%GS250 ml	WM	14 d	2,3
Gu JX 2001	34/30	45/19	E:58.43 ± 17.32 C:57.96 ± 17.14	E:15.16 ± 6.84 C:15.38 ± 7.02	SMI30 ml+WM, ivgtt, qd	5%GS250 ml	WM	14 d	1,3
Lu YH 2001	57/36	74/19	E:70.33 C:71.08	E:5–46 C:4–38	SMI30 ml+WM, ivgtt, qd	0.9%NS250 ml	WM	10 d	1,2,6
Jiang D 2002	25/25	37/13	E:69 C:67	NR	SMI40 ml+WM, ivgtt, qd	5%GS250 ml	WM	15 d	2
Sun TY 2003	52/48	59/41	E:58.3 C:59.2	NR	SMI50 ml+WM, ivgtt, qd	5%GS150 ml	WM	14 d	3
Hu YZ 2003	33/33	41/25	E:44–70 C:40–76	NR	SMI40 ml+WM, ivgtt, qd	5%GS250 ml	WM	14 d	1,6
Jiang WJ 2003	40/40	55/25	E:69.8 C:69.4	E:8–40 C:9–38	SMI40 ml+WM, ivgtt, qd	5%GS250 ml	WM	15 d	1
Shi B 2003	42/36	43/35	52–79	NR	SMI10~60 ml+WM, ivgtt, qd	5%GS250 ml	WM	7–10 d	1,6
Wu Q 2003	39/32	57/14	E:64.9 C:64.3	E:16.3 C:15.9	SM30 ml+WM, ivgtt, qd	5%GS250 ml	WM	10 d	1,6
Wang M 2004	31/31	42/20	E:68.5 C:69.5	NR	SMI40 ml+WM, ivgtt, qd	GS/0.9%NS250 ml	WM	15 d	3
Chen YL 2004	36/36	60/12	72 ± 3	NR	SMI50 ml+WM, ivgtt, qd	NR	WM	14 d	1,3,4
He HY 2005	23/21	31/13	E:62–74 C:61–76	E:8-12 C:8-11	SMI40 ml+WM, ivgtt, qd	5%GS250 ml	WM	14 d	1,6
Xue ZF 2005	34/30	43/21	E:76.2 C:74.8	NR	SMI50 ml+WM, ivgtt, qd	NR	WM	14 d	2,3
Zheng WT 2005	37/30	39/28	E:68.53 ± 6.21 C:69.56 ± 6.35	NR	SMI30 ml+WM, ivgtt, qd	5%GS250 ml	WM	7 d	1
Li B 2006	32/32	42/22	62	8-30	SMI40 ml+WM, ivgtt, qd	5%GS250 ml	WM	10 d	1
Chen LQ 2006	30/28	32/26	E:66–79 C:65–80	NR	SMI50 ml+WM, ivgtt, qd	5%GS200 ml	WM	7 d	1
Zheng QW 2006	60/60	68/52	E:74 C:69	E:25 C:24	SMI40 ml+WM, ivgtt, qd	5%GS200 ml	WM	14 d	2
Huo XL 2007	30/30	35/25	E:61 C:62	NR	SMI50 ml+WM, ivgtt, qd	5%GS250 ml	WM	10 d	2,3,6
Li HN 2008	63/50	66/47	E:68.5 C:69.1	NR	SMI45 ml+WM, ivgtt, qd	0.9%NS150 ml	WM	7–14 d	1
Cai L 2008	40/40	48/32	E:72.2 ± 1.2 C:71.1 ± 1.8	NR	SMI50 ml+WM, ivgtt, qd	NR	WM	14 d	2
Wang SH 2008	40/40	57/23	E:74 ± 2 C:73 ± 3	E:8–20 C:5–19	SMI50 ml+WM, ivgtt, qd	5%GS/0.9%NS250 ml	WM	15 d	1,6
Li HM 2008	61/54	76/39	E:52–89 C:53–87	E:17.9 ± 5.1 C:17.4 ± 5.3	SMI60 ml+WM, ivgtt, qd	5%GS250-500 ml	WM	15 d	2,4
Guo CD 2008	32/30	41/21	E:57.8 C:57.5	NR	SMI40-60 ml+WM, ivgtt, qd	5%GS250 ml	WM	14 d	1
Wan Q 2009	60/60	68/52	E:48–84 C:48–80	E:5–50 C:4–49	SMI40 ml+WM, ivgtt, qd	5%GS200 ml	WM	14 d	1,6
Xiao GZ 2009	38/37	40/35	E:70 ± 8 C:70 ± 8	E:5-20	SMI30~45 ml+WM, ivgtt, qd	5%GS250 ml	WM	14 d	1,6
Xie YB 2009	38/36	44/30	E:65.5 C:64.9	E:10.2 C:9.8	SMI100 ml+WM, ivgtt, qd	NR	WM	10 d	2,3
Zhao YH 2011	40/40	39/41	E:69.15 ± 11.00 C:64.45 ± 9.85	NR	SMI40 ml+WM, ivgtt, qd	5%GS200 ml	WM	14 d	1,2
Lv GM 2011	50/50	99/1	E:56–85 C:56–84	E:10–50 C:10–50	SMI50 ml+WM, ivgtt, qd	5%GS200 ml	WM	14 d	1,6
Song B 2011	24/24	29/19	45–75	NR	SMI50 ml+WM, ivgtt, qd	5%GS200 ml	WM	14 d	1
Wang H 2011	56/56	64/48	40–82	6–32	SMI60 ml+WM, ivgtt, qd		WM	15 d	1
Xu LN 2011	46/44	55/35	E:57. 4 C:56. 9	NR	SMI60-100 ml+WM, ivgtt, qd	5%GS/0.9%NS 100–200 ml	WM	10 d	1,6
Yin FJ 2011	20/20	26/14	E:58–52 C:NR	NR	SMI40 ml+WM, ivgtt, qd	5%GS200 ml	WM	14 d	1,6
Li SY 2012	30/31	36/25	E:67 ± 8.3 C:69 ± 9.1	E:11 ± 5.8 C:11 ± 7.8	SMI40 ml+WM, ivgtt, qd	5%GS250 ml	WM	10 d	1,6
Liu BH 2012	35/35	43/27	66.8 ± 11.4	NR	SMI40 mg+WM, ivgtt, qd	5%GS250 ml	WM	10–14 d	1
Han DX 2012	38/36	47/27	47–81	NR	SMI50 ml+WM, ivgtt, qd	5%GS250–500 ml	WM	7–14 d	1,6
Ye YL 2012	30/30	37/23	E:56.2 ± 5.3 C:56.5 ± 6.2	E:5–10 C:4–12	SMI30 ml+WM, ivgtt, qd	5%GS250 ml	WM	14 d	1,3
Wang LM 2012	21/21	30/12	54	0.5–14	SMI50 ml+WM, ivgtt, qd	5%GS200 ml	WM	14 d	1
Chen ZX 2013	25/25	27/23	E:59.3 ± 5.2 C:61.4 ± 5.5	E:4.7 ± 1.9 C:4.4 ± 2.3	SMI50 ml+WM, ivgtt, qd	5%GS250 ml	WM	14 d	1,4,5
Ni XZ 2013	35/35	43/27	E:66.82 ± 10.31 C:65.95 ± 10.52	E:10.83 ± 4.91 C:10.86 ± 4.96	SMI40 ml+WM, ivgtt, qd	5%GS250 ml	WM	10 d	1,6
Jin F 2013	28/28	31/25	E:52–86 C:48–81	E:5–32 C:4–30	SMI30 ml+WM, ivgtt, qd	5%GS250 ml	WM	14 d	1
Ma CH 2014	60/60	59/61	E:67 C:67	NR	SMI30 ml+WM, ivgtt, qd	5%GS250 ml	WM	14 d	1
Mei Y 2014	50/50	76/24	65.2 ± 5.3	15.3 ± 5.6	SMI60 ml+WM, ivgtt, qd	5%GS100 ml0.9%NS1,000 ml for diabetic	WM	10 d	1
Lei SC 2014	60/60	72/48	E:62.0 ± 1.0 C:62.5 ± 1.0	E:11.3 ± 1.6 C:11.2 ± 1.5	SMI50 ml+WM, ivgtt, qd	5%GS250 ml	WM	28 d	1,2,6
Zhu JF 2015	38/38	45/31	E:74.3 ± 2.5 C:73.1 ± 2.1	NR	SMI100 ml+WM, ivgtt, qd	0.9%NS100 ml	WM	14 d	1
Li F 2015*	30/32	27/35	E:45–65 C:42–68	E:4–18 C:3–16	SMI40 ml+WM, ivgtt, qd	5%GS250 ml	WM	10 d	1,2
Wang XM 2016*	30/30	43/17	E:66.50 ± 6.43 C:68.90 ± 7.01	NR	SMI60 ml+WM, ivgtt, qd	5%GS250 ml	WM	14 d	1,6
Yang J 2017	62/62	72/52	E:59.2 ± 9.8 C:60.0 ± 9.9	NR	SMI20 ml+WM, ivgtt, qd	5%GS250 ml	WM	14 d	3
Shi JP 2017	40/40	44/36	E:62.6 ± 11.2 C:61.9 ± 10.7	E:9–29 C:8–32	SMI40 ml+WM, ivgtt, qd	5%GS250 ml	WM	14 d	1
Li X 2004	38/35	41/32	63.5	14	SQFZI250 ml+WM, ivgtt, qd	/	WM	10–14 d	1,2,3
Cui HY 2010	26/26	30/22	E:66.48 ± 11.35 C:66.28 ± 11.19	E:8–10 C:9–15	SQFZI250 ml+WM, ivgtt, qd	/	WM	15 d	1,2,3,4
Luo WH 2010	65/61	68/58	E:60.3 ± 12.1 C:59.8 ± 11.5	E:7–20 C:8–18	SQFZI250 ml+WM, ivgtt, qd	/	WM	21 d	1

*: the RCT performed syndrome differentiation; C, control group; CWJI, Ciwujia injection (20 ml = 7 mg, 300–500 mg/time with 5–10%GS 250–500 ml, qd/bid); DZHJTI, Dazhuhongjingtian injection (10 ml/time with 5%GS 250 ml); E, experimental group; F, female; GS, dextrose solution; HQI, Huangqi injection (10–20 ml/time, qd); M, male; NR, not relate; NS, normal saline; SFI, Shenfu injection (20–100 ml/time with 5–10%GS 250–500 ml); SI, Shengmai injection (20–60 ml/time with 5%GS 250–500 ml); SMI, Shenmai injection (20–100 ml/time with 5%GS 250–500 ml); SQFZI, ShenqiFuzheng injection (qd). Outcomes: 1: the clinical effectiveness rate; 2: arterial blood gas analysis; 3: hemorheology; 4: hemodynamic; 5: right ventricular dimension: heart rate; 6: ADRs/ADEs.

### Methodological Quality

The eligible RCTs were performed using randomization; however, only seven RCTs stated their specific random method, and these studies were evaluated as “low risk”. One of the eligible RCTs claimed a double-blind design and was classified as “low risk” regarding performance bias. In addition, two RCTs were conducted in a single-blind manner and were assessed as “high risk” regarding performance bias because the color and usage of the Chinese herbal injections increased the potential for blinding to be broken. The remaining RCTs were evaluated as “unclear risk” for their selection, performance and detection bias due to insufficient information. Concerning attribution bias, all of the eligible RCTs provided complete data, indicating that they were “low risk”. In addition, one RCT did not report all outcomes in accord with its design and was assessed as “high risk”. The others were deemed “low risk”. In addition, other biases were also found, namely, whether a significant difference existed between the experimental and control groups. Fifteen of the eligible RCTs did not report the baseline, which may have had an impact on the results; thus, these 15 RCTs were evaluated as “high risk”. The others were considered “low risk” (**Presentation file**).

### Network Meta-Analysis

#### The Clinical Effectiveness Rate

A total of 98 RCTs reported the clinical effectiveness rate (Ciwujia injection, three RCTs; Dazhuhongjingtian, one RCT; Huangqi injection, 28 RCTs; Shenfu injection, nine RCTs; Shengmai injection, 13 RCTs; Shenmai injection, 41 RCTs; Shenqi Fuzheng injection, three RCTs). **Table 3** demonstrates the OR of this NMA, indicating that the combination of an eligible Chinese herbal injection and WM was superior to WM alone. The ORs of the comparisons were significantly different as follows: Ciwujia injection+WM vs. WM (OR = 0.27, 95% CI: 0.13–0.55), Dazhuhongjingtian injection+WM vs. WM (OR = 0.35, 95% CI: 0.11–0.91), Huangqi injection+WM vs. WM (OR = 0.23, 95% CI: 0.18–0.29), Shenfu injection+WM vs. WM (OR = 0.21, 95% CI: 0.12–0.35), Shengmai injection+WM vs. WM (OR = 0.29, 95% CI: 0.22–0.39), Shenmai injection+WM vs. WM (OR = 0.24, 95% CI: 0.20–0.30), and Shenqi Fuzheng injection+WM vs. WM (OR = 0.25, 95% CI: 0.11–0.54).

**Table 3:** Odds ratio/mean difference (95% CIs) of the various interventions.

Intervention	Clinical Effectiveness Rate (OR)	Partial Pressure of Arterial Oxygen (MD)	Partial Pressure of Arterial Carbon Dioxide (MD)	Level of Whole Blood Viscosity (MD)	Level of Fibrinogen (MD)	Mean Pulmonary Arterial Pressure (MD)	Right Ventricular Dimension (MD)
Ciwujia injection+WM *vs.*							
Dazhuhongjingtian injection+WM	0.78(0.23,2.94)	0.84(−3.52,4.73)	−0.29(-5.06,4.49)	–	–	–	–
Huangqi injection+WM	0.86(0.39,1.76)	−0.89(−3.50,1.73)	0.30(−3.44,4.15)	–	–	–	–
Shenfu injection+WM	0.61(0.19,2.33)	−0.44(−3.27,2.47)	0.18(−3.96,4.33)	–	–	–	–
Shengmai injection+WM	1.09(0.50,2.28)	−0.80(−4.78,3.27)	−0.58(-5.04,3.93)	–	–	–	–
Shenmai injection+WM	0.90(0.43,1.91)	−0.66(−3.19,1.87)	−0.01(−3.70,3.75)	–	–	–	–
Shenqi Fuzheng injection+WM	0.92(0.32,2.60)	−0.34(−5.30,4.24)	−0.27(−5.62,5.48)	–	–	–	–
WM	0.27(0.13,0.55)	1.75(−0.74,4.26)	−0.97(−4.67,2.70)	–	–	–	–
Dazhuhongjingtian injection+WM *vs.*							
Huangqi injection+WM	0.66(0.25,2.18)	−0.09(−3.64,3.20)	0.05(−3.28,3.31)	–	–	–	–
Shenfu injection+WM	0.61(0.19,2.33)	−0.35(−3.38,3.84)	−0.09(−3.81,3.66)	–	–	–	–
Shengmai injection+WM	0.83(0.31,2.73)	−0.08(−4.78,4.60)	−0.79(−4.91,3.12)	–	–	–	–
Shenmai injection+WM	0.69(0.26,2.29)	0.13(−3.37,3.38)	−0.26(−3.59,2.87)	–	–	–	–
Shenqi Fuzheng injection+WM	0.73(0.19,2.67)	0.39(−4.96,5.49)	−0.52(−5.57,4.70)	–	–	–	–
WM	0.35(0.11,0.91)	0.96(−2.27,4.43)	−0.71(−3.77,2.46)	–	–	–	–
Huangqi injection+WM *vs.*							
Shenfu injection+WM	1.07(0.62,2.00)	−0.45(−2.00, −1.06)	0.16(−2.07,2.35)	0.34(−9.17,9.86)	−0.15(−2.93,2.59)	−0.18(−17.88,15.45)	–
Shengmai injection+WM	0.79(0.54,1.14)	−0.08(−3.35,3.30)	0.87(−1.83,3.53)	–	0.04(−1.80,1.82)	−1.76(−18.20,14.90)	–
Shenmai injection+WM	0.95(0.69,1.29)	−0.22(−1.03,0.59)	0.33(−0.84,1.43)	0.84(−5.23,6.89)	1.24(−0.28,2.57)	3.43(−12.28,19.13)	1.19(−9.19,11.70)
Shenqi Fuzheng injection+WM	0.92(0.41,2.21)	−0.52(−4.44,3.70)	0.54(−3.80,4.57)	−0.34(−8.29,7.67)	2.48(−5.13,9.82)	3.39(−15.65,22.07)	–
WM	0.23(0.18,0.29)	0.87(0.15,1.55)	−0.67(−1.60,0.33)	−0.64(−6.35,5.04)	−0.29(−1.52,0.88)	−4.22(−16.16,7.77)	−3.82(−9.35,1.65)
Shenfu injection+WM *vs.*							
Shengmai injection+WM	0.73(0.39,1.28)	0.38(−3.07,4.04)	0.69(−2.44,3.93)	–	0.18(−2.67,3.02)	−0.61(−17.42,16.08)	–
Shenmai injection+WM	0.88(0.48,1.51)	0.24(−1.14,1.67)	0.17(−1.93,2.31)	0.49(−7.40,8.39)	1.36(−1.24,3.96)	4.58(−11.24,20.16)	–
Shenqi Fuzheng injection+WM	0.85(0.33,2.27)	−0.07(−4.08,4.21)	0.41(−4.27,4.88)	−0.68(−10.10,8.81)	2.62(−5.32,10.36)	4.41(−13.96,23.37)	–
WM	0.21(0.12,0.35)	1.32(0.02,2.71)	−0.80(−2.79,1.22)	−0.99(−8.56,6.59)	−0.15(−2.64,2.35)	−3.11(−14.98,9.05)	–
Shengmai injection+WM *vs.*							
Shenmai injection+WM	1.19(0.84,1.74)	−0.13(−3.59,3.12)	−0.55(−3.13,2.00)	–	1.19(−0.41,2.72)	5.14(−10.23,20.61)	–
Shenqi Fuzheng injection+WM	1.16(0.50,2.92)	−0.43(−5.59,4.71)	−0.36(−5.29,4.33)	–	2.45(−5.24,9.96)	5.08(−13.17,23.58)	–
WM	0.29(0.22,0.39)	0.95(−2.38,4.17)	−1.53(−3.99,0.99)	–	−0.34(−1.67,1.02)	−2.58(−13.93,9.21)	–
Shenmai injection+WM *vs.*							
Shenqi Fuzheng injection+WM	0.97(0.44,2.37)	−0.28(−4.18,3.79)	0.23(−4.01,4.15)	−1.19(−7.16,4.90)	1.28(−6.32,8.70)	−0.05(−17.68,17.50)	–
WM	0.24(0.20,0.30)	1.09(0.66,1.48)	−1.00(−1.52,-0.30)	−1.49(−3.62,0.72)	−1.52(−2.27,-0.69)	−7.60(−17.72,2.64)	–
Shenqi Fuzheng injection+WM *vs.*							
WM	0.25(0.11,0.54)	1.37(−2.68,5.25)	−1.20(5.08,3.01)	−0.29(−0.54,5.30)	−2.79(−10.11,4.76)	−7.52(−21.80,6.80)	–

Underlined results were statistically significant.

**Figure 2:**
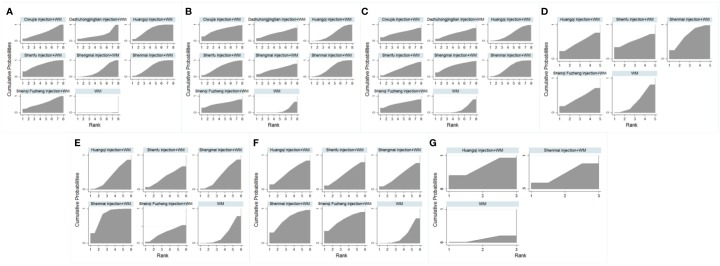
Surface under the cumulative ranking curve plots for all the different outcome interventions. **(A)** The Clinical Effectiveness Rate; **(B)** Partial Pressure of Arterial Oxygen; **(C)** Partial Pressure of Arterial Oxygen Carbon Dioxide; **(D)** The Level of Whole Blood Viscosity; **(E)** The Level of Fibrinogen; **(F)** Mean Pulmonary Arterial Pressure; **(G)** Right Ventricular Dimension.

Rankings of the analysis results are illustrated in **Table 4** and **Figure 2**, suggesting that Shenfu injection+WM had the highest clinical effectiveness rate, with a probability of 74.4%. Huangqi injection+WM (70.4%) was the second highest, and Shenmai injection+WM (63.0%) was the third highest.

**Table 4:** Ranking probabilities of the various interventions (%).

Intervention	Clinical Effectiveness Rate	Partial Pressure of Arterial Oxygen	Partial Pressure of Arterial Carbon Dioxide	Level of Whole Blood Viscosity	Level of Fibrinogen	Mean Pulmonary Arterial Pressure	Right Ventricular Dimension
Ciwujia injection+WM	53.1	**69.1**	52.7	–	–	–	–
Dazhuhongjingtian injection+WM	39.2	49.1	47.4	–	–	–	–
Huangqi injection+WM	70.4	45.2	46.1	49.6	40.8	51.5	66.3
Shenfu injection+WM	**74.4**	62.3	50.3	53.6	37.2	45.5	–
Shengmai injection+WM	40.4	48.5	**67.9**	–	42.2	41.9	–
Shenmai injection+WM	63	55.8	59.3	**69.1**	**82.2**	**71.2**	**73.3**
Shenqi Fuzheng injection+WM	59.2	56.6	56.9	44.6	72.2	67.7	–
WM	0.2	13.5	19.5	33.2	25.3	22.2	10.5

A warmer color indicates a higher ranking probability.

#### Arterial Blood Gas Analysis

The arterial blood gas analysis of the partial arterial oxygen and partial pressure of arterial carbon dioxide were subjected to meta-analysis. Both were measured in 30 RCTs (Ciwujia injection, two RCTs; Dazhuhongjingtian, one RCT; Huangqi injection, six RCTs; Shenfu injection, four RCTs; Shengmai injection, two RCTs; Shenmai injection,13 RCTs; and Shenqi Fuzheng injection, two RCTs). Huangqi injection+ WM (MD = 0.87, 95% CI: 0.15–1.55), Shenfu injection+ WM (MD = 3.88, 95% CI: 1.10–8.05) and Shenmai injection+ WM (MD = 1.09, 95% CI: 0.66–1.48) were significantly different from WM alone in boosting the partial pressure of arterial oxygen (**Table 3**).

Ranking analysis revealed that Ciwujia injection+WM was the optimal combination with a probability of 69.1%, and the other beneficial interventions were Shenfu injection+WM (62.3%) and Shenqi Fuzheng injection+WM (56.6%) (**Table 4** and **Figure 2**).

Shenmai injection+WM (MD = −1.00, 95% CI: −1.52- −0.30) was significantly different from WM alone in reducing the partial pressure of arterial carbon dioxide (**Table 3**).

Ranking analysis showed that Shengmai injection+WM was the optimal combination with a probability of 67.9%. Other beneficial interventions were Shenfu injection+WM (59.3%) and Shenqi Fuzheng injection+WM (56.9%) (**Table 4** and **Figure 2**).

#### Hemorheology

The hemorheology index, which includes whole blood viscosity and fibrinogen levels, were evaluated in this NMA. Nine RCTs tested whole blood viscosity levels (Huangqi injection, one RCT; Shenfu injection, one RCT; Shenmai injection, six RCTs; and Shenqi Fuzheng injection, one RCT). No significant differences were observed amongst the various interventions (**Table 3**).

Ranking analysis demonstrated that Shenmai injection+WM performed well in decreasing the whole blood viscosity level, with a probability of 69.1% (**Table 4** and **Figure 2**).

Fourteen RCTs tested fibrinogen levels (Huangqi injection, three RCT; Shenfu injection, one RCT; Shengmai injection, one RCT; Shenmai injection, eight RCTs; and Shenqi Fuzheng injection, one RCT). Shenmai injection+WM (MD = −1.52, 95% CI: −2.27 −0.69) was significantly different from WM alone with respect to lowering fibrinogen levels (**Table 3**).

The ranking analysis demonstrated that Shenmai injection+WM was more efficacious than the other treatments, with a probability of 82.2% (**Table 4** and **Figure 2**).

#### Hemodynamics

The hemodynamic index of this NMA focused on the mean pulmonary arterial pressures measured in seven RCTs (Huangqi injection, one RCT; Shenfu injection, one RCT; Shengmai injection, one RCT; Shenmai injection, three RCTs; and Shenqi Fuzheng injection, one RCT). No significant difference was observed between the various interventions (**Table 3**).

In the ranking analysis, Shenmai injection+WM was more effective in decreasing mean pulmonary arterial pressures with a probability of 71.2% (**Table 4** and **Figure 2**).

#### Right Ventricular Dimension

Five RCTs reported right ventricular dimensions (Huangqi injection, four RCTs and Shenmai injection, one RCT). None of the treatments produced significant decreases in right ventricular dimensions (**Table 3**).

Based on SUCRA, the ranking analysis revealed that Shenmai injection+WM could achieve a better impact in this outcome with a probability of 73.3% (**Table 4** and **Figure 2**).

### Cluster Analysis

**Figure 3:**
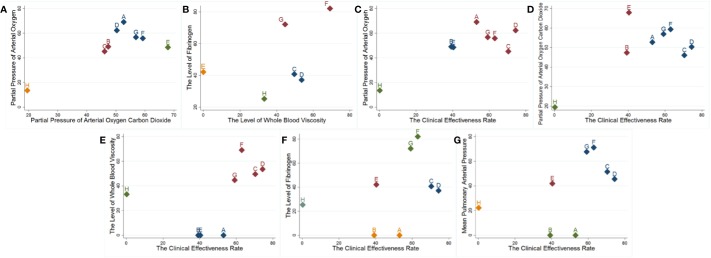
Cluster analysis plots. **(A, B)** Arterial blood gas analysis; **(C–G)** Cluster analysis on the clinical effectiveness rate and other outcomes. A: Ciwujia injection+WM; B: Dazhuhongjingtian injection+WM; C: Huangqi injection+WM; D: Shenfu injection+WM; E: Shengmai injection+WM; F: Shenmai injection+WM; G: Shenqi Fuzheng injection+WM.

The cluster analysis based on SUCRA is illustrated in **Figure 3**. First, the cluster analysis was conducted on arterial blood gas analysis. Among the eligible treatments, Shenmai injection+WM and Shenqi Fuzheng injection+WM achieved superior effects over the others in improving arterial blood gas levels, and WM alone ranked towards the bottom. Next, cluster analyses were performed on the clinical effectiveness rate and other outcomes. The results revealed that Shenfu injection+WM, Shenmai injection+WM, and Shenqi Fuzheng injection+WM were the highest ranked amongst the eligible interventions.

### Publication Bias

**Figure 4:**
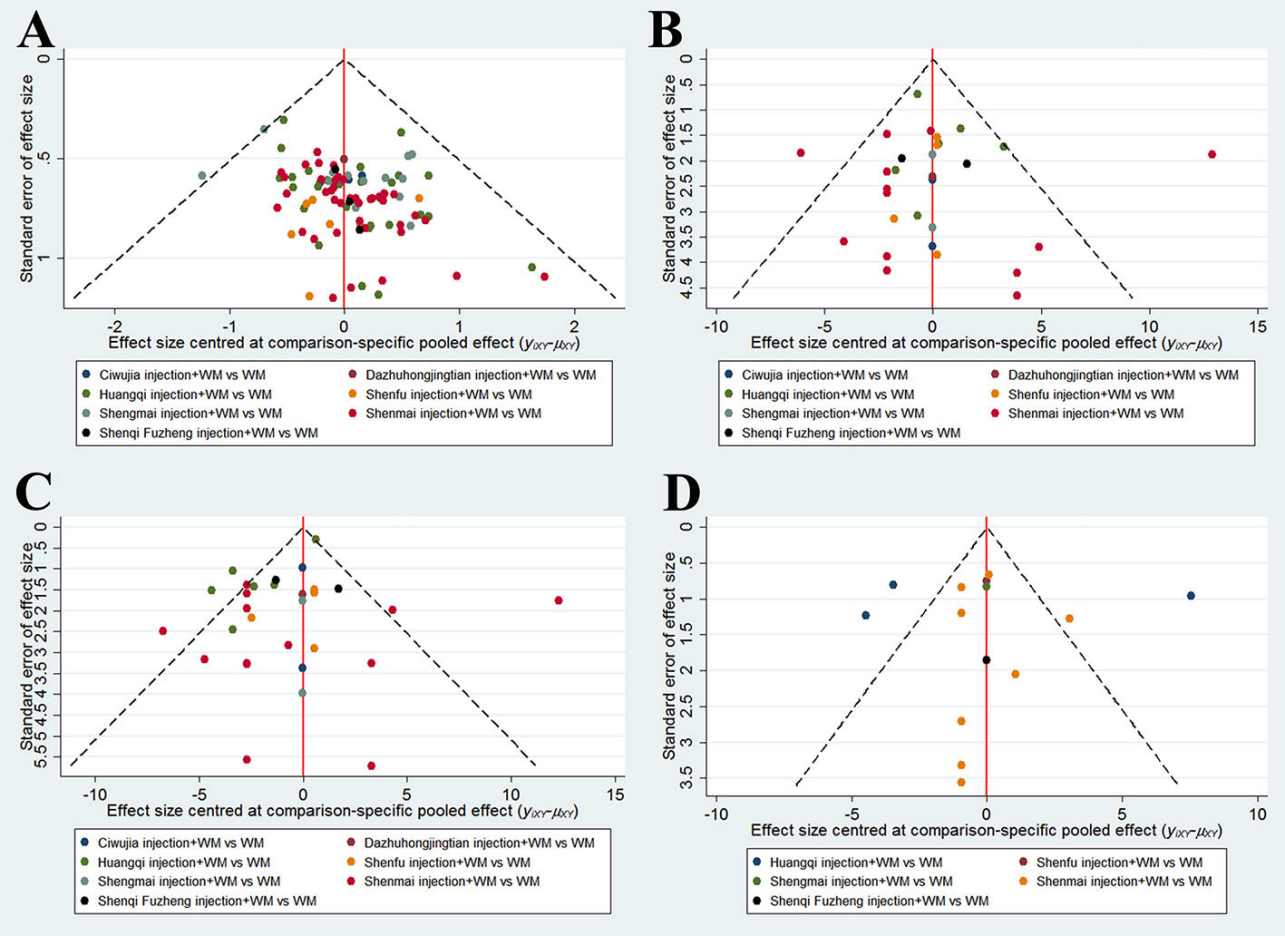
Funnel plots. **(A)** The Clinical Effectiveness Rate; **(B)** Partial Pressure of Arterial Oxygen; **(C)** Partial Pressure of Arterial Oxygen Carbon Dioxide; **(D)** The Level of Fibrinogen.

Publication bias was detected by funnel plots for outcomes included in more than 10 RCTs. Visual inspections showed that the eligible RCTs showed symmetry in the funnel plot of the clinical effectiveness rate, whereas the funnel plots for arterial blood gas analysis and fibrinogen levels were distributed asymmetrically and were out of line. Hence, a potential publication bias did exist (**Figure 4**).

### Adverse Drug Reactions (ADRs)/Adverse Drug Events (ADEs)

Amongst the eligible RCTs, 27 RCTs did not observe any ADRs/ADEs during the treatment, and 11 RCTs recorded ADRs/ADEs. Moreover, the remaining RCTs did not report ADRs/ADEs in their studies. Amongst the 11 RCTs, the intervention of the control group was WM, and three administered Huangqi injection+WM in the treatment group. In Zhu's research, three fever cases occurred in the treatment group. In Yin's research, nausea occurred in the treatment group. In Chen's research, one low fever case occurred in the treatment group. All of the above mentioned symptoms spontaneously resolved. One RCT reported two cases of xerostomia in the Shenmai injection+WM treatment group, which resolved with a slower drip rate. Seven RCTs reported ADRs/ADEs in the Shenmai injection+WM treatment group. In Wan's and Yin's research studies, the treatment group experienced two cases of xerostomia; in Xiao's research, three cases experienced pain at the injection site, and two cases experienced dizziness and palpitation in the treatment group; in Lv's research, one case of xerostomia and one case of tachycardia occurred in the treatment group; in Xu's research, one patient's heart rate decreased and two cases of headache, palpitation, and nausea in the corresponding control group were noted; in Lei's research, one case of rush and two cases of dizziness occurred in the treatment group, and three cases of rush, two cases of headache, and two cases of palpitations occurred in the corresponding control group; and in Wang's research, the treatment group and the control group reported one case of palpitations respectively. All of the aforementioned symptoms were relieved with a lower drip rate, which did not influence the RCTs.

## Discussion

The severity of PHD has been widely recognized due to its high mortality rates and heavy economic burden (Yun et al., 2016). Currently, a combination of Chinese herbal injections with WM have been reported to achieve better curative effects in PHD patients amongst various treatments, and its efficacy was verified by clinical trials and pairwise meta-analyses (Li et al., 2008; Shi et al., 2015; Wu et al., 2018). As the aim of this study was to discern the comparative effectiveness of Chinese herbal injections simultaneously, this NMA incorporated 118 RCTs, which included 10,085 patients, comparing the efficacy of seven Chinese herbal injections combined with WM versus WM alone, namely, Ciwujia injection+WM, Dazhuhongjingtian injection+WM, Huangqi injection+WM, Shenfu injection+WM, Shenmai injection+WM, Shenmai injection+WM, Shenqi Fuzheng injection+WM verse WM.

This NMA extensively evaluated these treatments and revealed that all eligible Chinese herbal injections plus WM had a positive effect in PHD patients. Three principal findings were observed as new evidence for the efficacy of Chinese herbal injections in treating PHD: (1) According to the OR/MD and cluster analysis results, all eligible Chinese herbal injections plus WM were superior to WM alone, particularly in promoting the clinical effectiveness rate, improving respiratory failure and reducing pulmonary arterial hypertension. (2) In contrast, Shenfu injection+WM, Shenmai injection+WM, and Shenqi Fuzheng injection+WM exhibited outstanding efficacies compared with the others. (3) It is essential for clinicians to pay more attention to drip rates during treatment. In addition, this NMA could not draw a specific conclusion regarding the safety of the Chinese herbal injections due to insufficient information.

In addition to pulmonary arterial hypertension, which is a precondition of PHD, limited respiratory and cardiac function and heart overload are predisposing factors (Tao and Zhang, 2004; Chen et al., 2013). Shenfu is a Chinese herbal medicine that is extracted from Hongshen (Ginseng radix et rhizoma rubra) and Fuzi (Aconm lateralis radix praeparaia) and functions by building up vital energy and relieving depletion. Although no experiments have shown that Shenfu injection could decrease pulmonary arterial hypertension, pharmacological experiments have already revealed that Shenfu injection has a specific influence on hemorheology. For instance, Shenfu injection is capable of lowering plasma viscosity, speeding blood flow velocity, alleviating platelet aggregation and relieving pulmonary artery thrombosis (Tao and Zhang, 2004). It also excels at alleviating bronchial smooth muscle spasms, protecting impaired lung tissue, and improving oxyhemoglobin saturation to enhance respiratory function (Sui, 2015). Moreover, Shenfu injection can modify the weak immune functions in PHD patients as well (Tian and Han, 2011; Sui, 2015). In addition, several pairwise meta-analyses showed that Shenfu injection plus WM improved the clinical effectiveness rate and respiratory and cardiac functions and lowered fibrinogen levels (Li et al., 2008). Shenmai contains Ginseng radix et rhizoma and Ophiopogonis radix and has an outstanding capacity to nourish and benefit (Cao et al., 2010). Pharmacological experiments confirmed that Shenmai injection had the ability to reduce pulmonary vascular resistance, improve the partial pressure of arterial oxygen, and enhance upper airway contractility through resisting upper airway fatigue, thus improving respiratory function (He et al., 2005; Li et al., 2006; Huang and Hu, 2017). It can also improve cardiac function by reducing the load on the heart and improving the oxygen supply for the myocardium (Li et al., 2006). With respect to immune function, Shenmai injection can boost CD3+, CD4+, and CD8+ T-lymphocyte levels (Sun, 2014; Huang and Hu, 2017). Shenqi Fuzheng contains Codonopsis radix and Astragali radix and is beneficial to strength and helpful in restoring vitality. No pharmacological experiments have verified its capacity in the lung, but its functions regarding positive inotropic effects, vasodilation and restraining heart failure have been confirmed (Wang and Zhang, 2016). Moreover, Shenqi Fuzheng injection enhanced the immune system *via* inhibition of T-lymphocytes (Liao and Xing, 2016).

In addition to the efficacy of Chinese herbal injections, their safety should also be considered. Though the occurrences of ADRs/ADEs in this NMA were low, approximately two-thirds of eligible RCTs did not report ADRs/ADEs, which meant their occurrence has not attracted clinical attention. While describing ADRs/ADEs, this NMA observed that an appropriate drip rate is essential in treatment. In addition, dosage, appropriate solution and syndrome differentiation should also be emphasized (Tan et al., 2014; Liu et al., 2016; Yang et al., 2018). This NMA has summarized this information (**Presentation file**).

This NMA was the first to apply a Bayesian model in the evaluation of Chinese herbal injection efficacy in the treatment of PHD to help in choosing a proper regimen. Bayesian NMA is considered the most applicable approach for a multiple-intervention NMA, as it enhances the relationship between the eligible RCTs and improves data utilization. In this NMA, a comprehensive literature search was performed to ensure the sample size of the NMA. Additionally, this NMA formulated strict eligibility criteria that control for the consistency between eligible RCTs on disease situations and interventions to reduce clinical heterogeneity. While the heterogeneity cannot be eliminated entirely, this NMA reduced it in this way. Notably, however, the pre-retrieval found that most relevant RCTs did not report the WM dosage; therefore, this NMA restricted the WM types and did not limit specific dosages. If the dosage description is included, then the quality of the NMA will be improved as well. Moreover, the kinds of outcomes varied because PHD involves lung and heart failure; therefore, on the basis of reading the clinical trials before the NMA was performed, seven representative outcomes that were measured in the highest number of studies were selected. This NMA used the clinical effectiveness rate to reflect the recovery condition of the PHD patients, arterial blood gas analysis to determine their functions on respiration, and hemorheology and hemodynamic index data to discern the pulmonary vasculature characteristics. Moreover, analyzing the right ventricular dimension reflected the right heart features.

Although the results of this NMA are promising, its limitations are worth mentioning. First, the eligible RCTs were conducted in China, and non-Chinese publications were excluded, resulting in a potential publication bias, which is illustrated in the funnel plot. Second, the credibility of this NMA was reduced because most eligible RCTs were carried out without adequate randomization, allocation and blinding. Third, the sample sizes of the outcomes need improvement. A small sample size may not detect a significant difference in comparisons. If the sample size is increased and the number of RCTs focused on different kinds of Chinese herbal injection was balanced, then the statistical strength of the data and the credibility of the NMA would be enhanced. In this context, further high quality and large scale RCTs are required to support this NMA.

## Conclusion

In general, this NMA performed a comprehensive evaluation and summary of Chinese herbal injections for treating PHD for the first time and proposed several findings. The results manifested that eligible Chinese herbal injections plus WM were superior to WM alone, especially Shenfu injection+WM, Shenmai injection+WM and Shenqi Fuzheng injection+WM. It is imperative for clinicians to incorporate the patients' symptoms and the Chinese herbal injections' efficacies when diagnoses are made. Larger sample sizes and high quality RCTs are needed to confirm and support this NMA.

## Data Availability Statement

All datasets presented in this study are included in the article/**Supplementary Material**.

## Author Contributions

JW and KW did conception and design of the network meta-analysis. KW, YW, XZe, MN, SL, ZM, and XZh performed the network meta-analysis. DZ, XD, and JW assessed the quality of the network meta-analysis. KW, XD, JW, YW, HW, and XZh analyzed study data. KW, XD, and HW wrote the paper. All authors read and approved the final version of the manuscript.

## Funding

This study received funding from the National Natural Science Foundation of China (No. 81473547 and No. 81673829) and the Young Scientists Training Program of Beijing University of Chinese Medicine.

## Conflict of Interest

The authors declare that the research was conducted in the absence of any commercial or financial relationships that could be construed as a potential conflict of interest.

The reviewer XL declared a shared affiliation, with no collaboration, with the authors to the handling editor at the time of review.

## Supplementary Material

Data Sheet 1 This file contains seven parts, which includes items regarding the PRISMA checklist for network meta-analysis and corresponding pages of this study, the search strategy of traditional Chinese medicine injections and English databases, information about the included randomized controlled trials and Chinese herbal injections, and a reference list of the eligible randomized controlled trials. This file also contains two figures, a flow chart of the included randomized controlled trial searches and a risk of bias graph.

## Abbreviations

95% CI, 95% credible interval; ADRs/ADEs, adverse drug reactions/adverse drug events; CHIs, Chinese herbal injections; PHD, Pulmonary heart disease; MD, mean difference; NMA, network meta-analysis; OR, odds ratio; RCTs, randomized controlled trials; WM, western medicine; SUCRA, surface under the cumulative ranking area curve.
