# Supplementary material for: Comparative Efficacy of Chinese Herbal Injections for Pulmonary Heart Disease: A Bayesian Network Meta-Analysis of Randomized Controlled Trials
Source: Front Pharmacol. 2020 May 7;11:634. doi: 10.3389/fphar.2020.00634 (PMC7224249; doi:10.3389/fphar.2020.00634)
Supplement: Data Sheet 1 — This file contains seven parts, which includes items regarding the PRISMA checklist for network meta-analysis and corresponding pages of this study, the search strategy of traditional Chinese medicine injections and English databases, information about the included randomized controlled trials and Chinese herbal injections, and a reference list of the eligible randomized controlled trials. This file also contains two figures, a flow chart of the included randomized controlled trial searches and a risk of bias graph. [file DataSheet_1.doc]

**Supplementary Material**

**Detailed information on Chinese herbal injections**

| **Chinese herbal injection** | **Source** | **Raw material** | **Phytochemical compositions** | **Chemical composition criteria** | **Therapeutic claims in TCM** | **Indications** | **Quality control reported? (Y/N)** | **Chemical analysis reported? (Y/N)** |
| --- | --- | --- | --- | --- | --- | --- | --- | --- |
| Ciwujia injection | Wandashan Pharmaceutical in Heilongjiang province | *ACANTHOPANACIS SENTICOSI RADIX ET RHIZOMA SEU CAULIS* (Manyprickle Acanthopanax) | Eleutheroside, Syringoside, Isofraxidin and et al. | The **total flavonoids** contained in this product are calculated as anhydrous Rutin (C_27_H_30_O_16_) and should be 90.0-110.0% of the labeled amount.  The labeled amount is: 20 ml of the injection contains 100 mg of total flavonoids; 100 ml of the injection contains 300 mg of total flavonoids; and 250 ml of the injection contains 500 mg of total flavonoids. | Moderate reinforcing liver and kidney, replenishing essence, toning up bones. | coronary heart disease, angor pectoris with neurasthenia, transient ischemic attack, cerebral arteriosclerosis, cerebral infarction caused by deficiency of the liver and kidney essence , etc. | Y - National Food and Drug Administration National Drug Standards | N |
| Dazhuhongjingtian injection | Tonghua Yusheng Pharmaceutical Co., Ltd. | *RHODIOLAE CRENULATAE RADIX ET RHIZOMA* (Rhodiola Rosea) | Salidroside, Rhodiosin, Rhodionin, Herbacetin, Kaempferol, Quercetin and et al. | This product contains 1 mg of **total sugar** in anhydrous glucose (C_6_H_12_O_6_), not less than 20.0mg.  Not less than 3.5mg per 1ml of **Salidroside** (C_14_H_20_O_7_), not less than 0.30mg of **Tyrosol** (C_8_H_10_O_2_). | Activating blood and dissolving stasis. | Stable angina pectoris and others caused by insufficient heart blood with the symptoms of stabbing pain and colic pain in chest, chest distress, palpitation, and thread pulse, etc. | Y - National Food and Drug Administration National Drug Standards | N |
| Huangqi injection | CHENGDU DIAO PHARMACEUTICAL GROUP Co., Ltd./ CHIATAI QINGCHUNBAO Pharmaceutical Co., Ltd./ Shanghai Fuda Pharmaceutical Drug manufacturing Co., Ltd. | *ASTRAGALI RADIX* (Mikvetch Root) | Astragaloside, Isoastragaloside, Acetylastragaloside, Astragaline, Benzofiiranoidlignan and et al. | Each 1ml of Huangqi injection contains not less than 0.08mg of **Astragaloside IV** (C_41_H_68_O_14_). | *Qi* tonifying and body strengthening resistance, heart nourishment, spleen fortification and dampness removal. | Insufficiency of the heart-*qi* and blood stasis syndrome of viral myocarditis, cardiac insufficiency and et al.; hepatitis with spleen deficiency and dampness syndrome, etc. | Y - National Food and Drug Administration National Drug Standards | N |
| Shenfu injection | Yaan Three Nine Pharmaceutical Co., Ltd. | *GINSENG RADIX ET RHIZOMA RUBRA* (Red Ginseng)*, ACONm LATERALIS RADIX PRAEPARAIA* (Prepared Common Monkshood Daughter Root) | Ginsenoside, Benzoyldeoxyaconitine, Benzoylhypaconitine, Benzoylaconitine, Benzoylmesaconitine and et al. | Each 1ml containing **total Ginsenoside Saponins** is not less than 0.5mg based on Ginsenoside Rb_1_ (C_54_H_92_O_23_). | Revives *yang* for resuscitation, *qi* tonifying and exhaustion prevention. | Desertion syndrome caused by excessive *yang*-*qi* insufficiency (infectious, haemorrhagic and fluid loss shock); *yang* or *qi* deficiencies with palpitations, cough, stomach ache, diarrhoea, rheumatism, etc. | Y - National Food and Drug Administration National Drug Standards | N |
| Shengmai injection | Jiangsu Suzhong Construction Group Co., Ltd./ Changshu LEI YUN SHANG Pharmaceutical Co., Ltd./ Shanxi TAIHANG Pharmaceutical Co., Ltd. | *GINSENG RADIX ET RHIZOMA RUBRA* (Red Ginseng)*, OPHIOPOGONIS RADIX* (Dawarf Lilyturf Tuber)*, SCHISANDRAE CHINENSIS FRUCTUS* (Chinese Magnoliavine Fruit) | Ginsenoside, Panaxatriol, Ophiopogonone, Methylophiopogonanone, Shikimic Acid and et al. | Each 1ml contains **Ginsenoside Rg1** not less than 0.08mg, containing **Ginsenoside Re** not less than 0.04mg. | Nourishing *qi* and *yin*, exhaustion prevention. | Palpitations, hard breathing, cold limbs and other symptoms caused by *qi* and *yin* insufficiencies, myocardial infarction, cardiogenic shock, cardiogenic shock and others with abovementioned symptoms, etc. | Y - National Food and Drug Administration National Drug Standards | N |
| Shenmai injection | CHIATAI QINGCHUNBAO Pharmaceutical Co., Ltd./ West China Medical University Pharmaceutical Factory/ Sichuan Sanjing Shenghe Pharmaceutical Ltd./ Yaan Three Nine Pharmaceutical Co., Ltd./ Hebei Shenwei Pharmaceutical Co., Ltd. | *GINSENG RADIX ET RHIZOMA RUBRA* (Red Ginseng)*,* 4g  *OPHIOPOGONIS RADIX* (Dawarf Lilyturf Tuber) 4g | Ginsenoside, Ophiopogonin, Ophiopogon Polysaccharide and et al. | The **total saponin** per 1ml is not less than 0.80 mg based on Ginsenoside Re (C_48_H_82_O_18_). | *Qi* tonifyng, exhaustion prevention, *yin* nourishment, body fluid generation, pulse activation. | Shock, coronary heart disease, viral myocarditis, chronic pulmonary heart disease and neutropenia with *qi* and *yin* deficiencies; improves immune function of patients with tumours, combined with chemotherapy to enhance curative effects and to reduce toxic and side effects, etc. | Y - National Food and Drug Administration National Drug Standards | N |
| Shenqi Fuzheng injection | LIVZON Pharmaceutical Group Co., Ltd. | *ASTRAGALI RADIX* (Mikvetch Root)*, CODONOPSIS RADIX* (Tangshen) | Astragaloside I, Astragaloside II, Astragaloside III, Astragaloside IV, Lobetyolin, Formononetin and et al. | Not less than 13.0 mg per 1 ml of **total solids**.  The **total saponin** per 1 ml is not less than 0.12 mg based on Astragaloside IV (C_41_H_68_O_14_).  Each 1 ml containing **Huangqi** is not less than 0.004 mg based on Astragaloside (C_41_H_68_O_14_).  The total **sugar** contained per 1 ml is not less than 3.5 mg based on anhydrous glucose (C6H12O6).  This product contains **sodium chloride (NaCl)** should be 98.0% -104.0% of the indicated amount. | *Qi* toning and *yin* nourishment*.* | Fatigue, lack of strength, vertigo caused by asthenia of pulmonosplenic *qi*; auxiliary treatment of the above symptoms for lung cancer and gastric cancer, etc. | Y - National Food and Drug Administration National Drug Standards | N |

**References**

Cao, L. M., Zhao, X., Liu, T. T., Tang, M., Li, Q., Pan, Y., et al. (2017). Simultaneous determination of five components in school of pharnacy, Shenfu injection with HPLC method. Pharm Clin Chin Mater Med. 7, 22-24.

Cao, M. M., Gao, Y. Y., Ma, Y. X., and Li, J. (2015). Research progress on the chemical composition of astragalus membranaceus and its protective effect on myocardial ischemia reperfusion injury. Inf Tradit Chin Med. 32, 120-123.

Fan, H. X. (2014). Pharmacokinetic study of the major active components of acanthopanax senticoccus. University of Jinan.

Gu, H. Y., Zhang, S. Y., Huang, W. H., Liu, X. H., Wang, Y., Fan, C. L., et al. (2013). Chemical constituents of shenqi fuzheng injection. Chin Tradit Pat Med. 35, 1494-1499.

Huang, J., Shao, Q., Xiang, X. H., Ge, Z. W., and Fan, X. H. (2014). Identification of phenylpropanoids in ciwujia injection by HPLC-MS. *China J Chin Mater Med.* 39, 2513-2520.

Li, N., Huang, X., Wang, B., Zhang, J. Y., Wang, H. Y., Dong, T. X., et al. (2017). Identification of chemical constituents of ophiopogon japonicus in shenmai injection. Chin Tradit Pat Med. 39, 2340-2344.

Liu, W. S. (2011). Study on HPLC fingerprint of red ginseng and shenfu injection. Shenyang Pharmaceutical University.

Shu, J., and Yang, S. B. (2016). Simultaneous determination of six components in shenqi fuzheng injection by HPLC. China Pharm. 27, 4295-4297.

Su, P., Lv, S. F., Fan, X. M, Liang, Q. L., Wang, Y. M., and Luo, G. A. (2011). Protective effect of shenmai injection and its effective components on H2 O2 induced myocardial cell injury. Chin Tradit Pat Med. 33, 2150-2154.

Wang, Q., Liu, Y., Zheng., X. W., Yu, J. D., Dai, Z., Lu, J., et al. (2012). Study on homoisoflavones in shengmai injection. Chin Pharm J. 47, 1539-1542.

Wang, Q., Zheng., X. W., Yu, J. D., Liu, Y., Dai, Z., Lu, J., et al. (2012). Study on saponins in shengmai injection. China J Chin Mater Med. 18, 1731-1734.

Wu, J. G., Dong, L., Chen, H. J., and Xu, X. P. (2017). Determination of shikimic acid in shengmai injection by HPLC. Chin Measur Test Technol. 43, 41-44.

Yang, R. J. (2012). Study on chemical constituents of shenfu injection. Jilin University.

Zhang, S. Y., Fan, C. L., Wang, L., Liu, X. H., Sun, X. W., and Ye, W. C. (2011). Chemical constituents of shenqi fuzheng injection. Chin Tradit Pat Med. 33, 1743-1748. 3. Zhao, L. (2017). Determination of the active ingredients of rhodiola extract and the effect of hypoxia on the pharmacokinetics. Capital Institute of Medicine.

Zhao, L., Qi, T., Hui, B. K., Xu, P. X., and Xue, M. (2016). Quantification of six active ingredients in rhodiola extract by UPLC-MS/MS. J Int Pharm Res. 43, 975-979.

Zhao, Y. D. (2016). Chemical composition of astragalus injection and its systematic evaluation on cerebral infarction. Beijing University of Traditional Chinese Medicine.

**PRISMA checklist for network meta-analysis:**

| **Section/topic** | **#** | **Checklist item** | **Reported on page #** |
| --- | --- | --- | --- |
| **TITLE** | | |  |
| Title | 1 | Identify the report as a systematic review incorporating a network meta-analysis (or related form of  meta-analysis). | 1 |
| **ABSTRACT** | | |  |
| Structured summary | 2 | Provide a structured summary including, as applicable:  Background: main objectives  Methods: data sources; study eligibility criteria, participants, and interventions; study appraisal; and synthesis methods, such as network meta-analysis.  Results: number of studies and participants identified; summary estimates with corresponding confidence/credible intervals; treatment rankings may also be discussed. Authors may choose to summarize pairwise comparisons against a chosen treatment included in their analyses for brevity.  Discussion/Conclusions: limitations; conclusions and implications of findings.  Other: primary source of funding; systematic review registration number with registry name. | 2 |
| **INTRODUCTION** | | |  |
| Rationale | 3 | Describe the rationale for the review in the context of what is already known, including mention of why a network meta-analysis has been conducted | 2-3 |
| Objectives | 4 | Provide an explicit statement of questions being addressed with reference to participants, interventions, comparisons, outcomes, and study design (PICOS). | 2-3 |
| **METHODS** | | |  |
| Protocol and registration | 5 | Indicate if a review protocol exists and if and where it can be accessed (e.g., Web address), and, if available, provide registration information including registration number. | / |
| Eligibility criteria | 6 | Specify study characteristics (e.g., PICOS, length of follow-up) and report characteristics (e.g., years considered, language, publication status) used as criteria for eligibility, giving rationale. Clearly describe the eligible treatments included in the treatment network, and note whether any have been clustered or merged into the same node (with justification). | 3-4 |
| Information sources | 7 | Describe all information sources (e.g., databases with dates of coverage, contact with study authors to identify additional studies) in the search and date last searched. | 3 |
| Search | 8 | Present full electronic search strategy for at least one database, including any limits used, such that it could be repeated. | 3 |
| Study selection | 9 | State the process for selecting studies (i.e., screening, eligibility, included in systematic review, and, if applicable, included in the meta-analysis). | 4 |
| Data collection process | 10 | Describe method of data extraction from reports (e.g., piloted forms, independently, in duplicate) and any processes for obtaining and confirming data from investigators. | 4 |
| Data items | 11 | List and define all variables for which data were sought (e.g., PICOS, funding sources) and any assumptions and simplifications made. | 3-4 |
| Geometry of the network | 12 | Describe methods used to explore the geometry of the treatment network under study and potential biases related to it. This should include how the evidence base has been graphically summarized for presentation, and what characteristics were compiled and used to describe the evidence base to readers | 5 |
| Risk of bias within individual studies | 13 | Describe methods used for assessing risk of bias of individual studies (including specification of whether this was done at the study or outcome level), and how this information is to be used in any data synthesis. | 4-5 |
| Summary measures | 14 | State the principal summary measures (e.g., risk ratio, difference in means). Also describe the use of additional summary measures assessed, such as treatment rankings and surface under the cumulative ranking curve (SUCRA) values, as well as modified approaches used to present summary findings from meta-analyses. | 5 |
| Planned methods of analysis | 15 | Describe the methods of handling data and combining results of studies for each network meta-analysis. This should include, but not be limited to: Handling of multigroup trials; Selection of variance structure; Selection of prior distributions in Bayesian analyses; and Assessment of model fit. | 5 |
| Assessment of inconsistency | 16 | Describe the statistical methods used to evaluate the agreement of direct and indirect evidence in the treatment network(s) studied. Describe efforts taken to address its presence when found. | / |
| Risk of bias across studies | 17 | Specify any assessment of risk of bias that may affect the cumulative evidence (e.g., publication bias, selective reporting within studies) | 5 |
| Additional analyses | 18 | Describe methods of additional analyses if done, indicating which were pre-specified. This may include, but not be limited to, the following: Sensitivity or subgroup analyses; Meta-regression analyses; Alternative formulations of the treatment network; and Use of alternative prior distributions for Bayesian analyses (if applicable). | 5 |
| **RESULTS** | | |  |
| Study selection | 19 | Give numbers of studies screened, assessed for eligibility, and included in the review, with reasons for exclusions at each stage, ideally with a flow diagram. | 5 |
| Presentation of network structure | 20 | Provide a network graph of the included studies to enable visualization of the geometry of the treatment network. | 5-6 |
| Summary of network geometry | 21 | Provide a brief overview of characteristics of the treatment network. This may include commentary on the abundance of trials and randomized patients for the different interventions and pairwise comparisons in the network, gaps of evidence in the treatment network, and potential biases reflected by the network structure. | 6 |
| Study characteristics | 22 | For each study, present characteristics for which data were extracted (e.g., study size, PICOS, follow-up period) and provide the citations. | 6 |
| Risk of bias within studies | 23 | Present data on risk of bias of each study and, if available, any outcome level assessment. | 6 |
| Results of individual studies | 24 | For all outcomes considered (benefits or harms), present, for each study: 1) simple summary data for each intervention group, and 2) effect estimates and confidence intervals. Modified approaches may be needed to deal with information from larger networks. | 7-9 |
| Synthesis of results | 25 | Present results of each meta-analysis done, including confidence/credible intervals. In larger networks, authors may focus on comparisons versus a particular comparator (e.g., placebo or standard care), with full findings presented in an appendix. League tables and forest plots may be considered to summarize pairwise comparisons. If additional summary measures were explored (such as treatment rankings), these should also be presented. | 7-9 |
| Exploration for inconsistency | 26 | Describe results from investigations of inconsistency. This may include such information as measures of model fit to compare consistency and inconsistency models, P values from statistical tests, or summary of inconsistency estimates from different parts of the treatment network. | / |
| Risk of bias across studies | 27 | Present results of any assessment of risk of bias across studies for the evidence base being studied. | 7-9 |
| Results of additional analyses | 28 | Give results of additional analyses, if done (e.g., sensitivity or subgroup analyses, meta-regression  analyses, alternative network geometries studied, alternative choice of prior distributions for  Bayesian analyses, and so forth). | 8 |
| **DISCUSSION** | | |  |
| Summary of evidence | 29 | Summarize the main findings, including the strength of evidence for each main outcome; consider their relevance to key groups (e.g., health care providers, researchers, and policymakers). | 9-11 |
| Limitations | 30 | Discuss any limitations at the study and outcome level (e.g., risk of bias), and at the review level (e.g., incomplete retrieval of identified research, reporting bias). Comment on the validity of the assumptions, such as transitivity and consistency. Comment on any concerns regarding network geometry (e.g., avoidance of certain comparisons). | 11 |
| Conclusions | 31 | Provide a general interpretation of the results in the context of other evidence, and implications for future research. | 11 |
| **FUNDING** | | |  |
| Funding | 32 | Describe sources of funding for the systematic review and other support (e.g., supply of data); role of funders for the systematic review. This should also include information regarding whether funding has been received from manufacturers of treatments in the network and/or whether some of the authors are content experts with professional conflicts of interest that could affect use of treatments in the network. | 12 |

Search strategy

| 1. **Pubmed** |
| --- |
| #1 randomized controlled trial [Publication Type]  #2 controlled clinical trial [Publication Type]  #3 random* [All Fields]  #4 #1 OR #2 OR #3  #5 huangqi [Title/Abstract]  #6 astragalus [Title/Abstract]  #7 shengfu [Title/Abstract]  #8 shengmai [Title/Abstract]  #9 shenmai [Title/Abstract]  #10 shenqi fuzheng [Title/Abstract]  #11 yiqifumai [Title/Abstract]  #12 #5 OR #6 OR #7 OR #8 OR #9 OR #10 OR #11  #13 Pulmonary Heart Disease [MeSH Terms]  #14 Pulmonary Heart Disease* [Title/Abstract]  #15 Cor Pulmonale [Title/Abstract]  #16 #13 OR #14 OR #15  #17 #4 AND #12 AND #16 |
| 2. **Cochrane Library** |
| #1 random*  #2 huangqi: ti,ab,kw  #3 astragalus: ti,ab,kw  #4 shengfu: ti,ab,kw  #5 shengmai: ti,ab,kw  #6 shenmai: ti,ab,kw  #7 shenqi fuzheng: ti,ab,kw  #8 yiqifumai: ti,ab,kw  #9 #2 OR #3 OR #4 OR #5 OR #6 OR #7 OR #8  #10 Pulmonary Heart Disease: MeSH  #11 #1 AND #9 AND #10 |
| 3. **EMBASE** |
| #1 Randomized Controlled Trial/exp  #2 huangqi/ti,ab,kw  #3 astragalus/ti,ab,kw  #4 shengfu/ti,ab,kw  #5 shengmai/ti,ab,kw  #6 shenmai/ti,ab,kw  #7 shenqi fuzheng/ti,ab,kw  #8 yiqifumai/ti,ab,kw  #9 #2 OR #3 OR #4 OR #5 OR #6 OR #7 OR #8  #9 Cor Pulmonale /exp  #10 Pulmonary Heart Disease* /ti,ab,kw  #11 #9 OR #10  #12 #1 AND #9 AND #11 |

**Reference list:**

Bai, R., Li, Q. Y., and Fang, X. J. (1999). Clinical observation of 25 cases of pulmonary heart disease with Huangqi injection. *J. Shanxi. Med. Coll. Contin. Educ.* 9, 39-40.

Cai, J., Li, X. P., and Chen, P. (2003). Membranous milkvetch root solution therapy for 56 patients with pulmonary heart disease and heart failure. *Chin. J. Med. Writing.* 10, 1097-1098.

Cai, L., and Shen, D. (2008). Clinical observation on the treatment of chronic pulmonary heart disease with Shengmai injection. *J. Zhejiang. Chin. Med. Univ.* 32, 360-361.

Cai, Z. W., and Nong, H. S. (1999). Clinical observation of Shenmai injection in the treatment of pulmonary heart disease. *Zhejiang. J. Integr. Tradit. Chin. West. Med.* 9. 132.

Chen, D. Z. (2000). Clinical effect of Shengmai injection on pulmonary heart disease. *J. Guiyang. Med. Coll.* 25, 416-417.

Chen, G. Y. (2007). Clinical observation of Shengmai injection in the treatment of acute episodes of pulmonary heart disease. *Chin. Med. Fact. Mine.* 21, 490-491.

Chen, L. Q., and Fu, Y. (2006). Effect of Shenmai injection on acute exacerbation of chronic pulmonary heart disease. *J. Chin. Med. Pharmacol.* 34, 56.

Chen, X. M., and Hao, H. Y. (2009). Clinical observation of 40 cases of Astragalus injection for pulmonary heart disease. *Med. J. Chin. People. Health.* 21, 1833-1834.

Chen, X. Z. (2002). Observation on the curative effect of Shengmai injection on acute stage of pulmonary heart disease. *Lishizhen. Med. Mater. Med. Res.* 13, 563.

Chen, Y. L., Ou, Y. M., and Zhong, J. R. (2004). Clinical effects and principle of Shenmai injection in the treatment of chronic cor pulmonale. *Acta. Acad. Med.* Jiangxi. 44, 59-62.

Chen, Z. J. (2010). Observation on the therapeutic effect of Shengmai injection in the adjuvant treatment of patients with acute exacerbation of chronic pulmonary heart disease. *Chin.J. New. Clin. Med.* 3, 864-865.

Cui, H. Y., Quan, G. L., Wang, C. L., Zeng, C., and Cai, S. N. (2010). Clinical observation on Shenqi Fuzheng injection for treatment of acute heart failure caused by chronic cor pulmonale. *Cardiovasc. Dis. J. Integr. Tradit. Chin. West. Med.* 8, 1012-1024.

Dong, X. F. (2007). Therapeutic effect of 87 cases of Shengmai injection for pulmonary heart disease. *Mod. J. Integr. Tradit. Chin. West. Med.* 16, 5297.

Fan, D. B., Qin, X. P., Bai, Y. H., Xu, J. Z., Zeng, Y. H., Zeng, G. Q., et al. (2009). Clinical observation of 62 cases of Shenfu injection for pulmonary heart disease. Academic Seminar on Wu Peiheng Academic Thought.

Fan, X. Y. (2003). Efficacy of Huangqi injection in treating severe pulmonary heart disease with respiratory failure. *Chin. J. Misdiagn.* 3, 1519-1520.

Gao, D. F. (1999). Effect of Astragalus injection on the level of superoxide dismutase in patients with pulmonary heart disease. *Chin. J. New. Drugs. Clin. Rem.* 18, 376-377.

Gao, Y. D. (2001). Observation on the efficacy of Shengmai injection in treating 42 cases of pulmonary heart disease.*J. Jinggangshan. Med. Coll.* 8, 62.

Gu, J. X., Dai, X. H., Wang, L., and Zhou, Y. X. (2001). Clinical observation of 34 cases of Shenmai injection for pulmonary heart disease. *Clin. J. Anhui. Tradit. Chin. Med.* 13, 336-338.

Guo, C. D. (2008). Therapeutic effect of Shenmai injection in the treatment of pulmonary heart failure. *Chin. J. Misdiagn.* 8, 4626-4627.

Guo, F. C., and Huang, F. C. (2014). Effect of Shenfu injection in the acute stage of pulmonary heart disease. *Med. Inf.* 27, 217.

Guo, H. D., Liu, L. H., Yuan, W. F., and Liao, Z. M. (2005). Therapeutic effect of Astragalus injection in treatment of chronic pulmonary heart disease combined with heart failure. *Pract. Clin. J. Integr. Tradit. Chin. West. Med.* 5, 22.

Guo, X. H. (1999). Efficacy of Shenmai injection in the treatment of pulmonary heart failure. *Chin. J. Integr. Tradit. West. Med. Intensive. Crit. Care.* 6, 501.

Han, D. X. (2012). Observation of Shenmai injection in treatment of chronic pulmonary heart disease. *J. Clin. Ration. Drug. Use.* 5, 59.

He, H. Y., and Li, D. G. (2005). Observation on the therapeutic effect of Shenmai injection in the treatment of chronic pulmonary heart disease. *Chin. J. Misdiagn.* 5, 2423-2424.

heart disease in 31 cases*. Fujian. Med. J.* 26, 172-173.

Hu, Y. Z. (2003). Clinical observation on 33 cases of chronic cardiopulmonary disease treated by Shenmai injection. *Hunan. Guiding. J. Tradit. Chin. Med.* Pharmacol. 9, 13-14.

Hui, S. L., and Hao, W. H. (2008). Efficacy of Astragalus injection in the treatment of chronic pulmonary heart disease. *Mod. J. Integr. Tradit. Chin. West. Med.* 17, 1965.

Huo, X. L. (2007). Clinical observation of 30 cases of chronic pulmonary heart disease complicated with respiratory failure by Shenmai injection. *Tianjin. Pharm.* 19, 41-42.

Jiang, D., and Tu, J. W. (2002). Clinical value of Shenmai injection in treating heart failure of pulmonary heart disease. *J. Emerg. Syndromes. Tradit. Chin. Med.* 11, 28-29.

Jiang, Q. F., and Xu, B. S. (1996). Therapeutic effect of Shenmai injection on chronic pulmonary heart disease. *Zhejiang. J. Integr. Tradit. Chin. West. Med.* 6, 86-87.

Jiang, W. J. (2003). Therapeutic effect of Shenmai injection in the treatment of pulmonary heart failure. *J. Emerg. Syndromes. Tradit. Chin. Med.* 12, 430.

Jin, F. (2013). Clinical observation of 28 cases of Shenmai injection on acute episode of chronic pulmonary heart disease. *Med. Front.* 3, 203-204.

Kong, X. M., Li, A. M., Hu, X. Y., Zhang, X. R., Xu, J. Y., and Du, Y. C. (2003). Clinical observation of Shenfu injection for pulmonary heart disease. *Shanxi. Med. J.* 32, 560-561,

Lei, S. C. (2015). Clinical observation of Shenmai injection for pulmonary heart disease. *Chin. Foreign Health. Abstr.* 10, 101-102.

Li, B. (2006). Clinical observation of 32 cases of chronic pulmonary heart failure treated by Shenmai injection. *Shandong. Med. J.* 46, 100.

Li, D. H., Chen, N., Li, H., Liang, H. Y., Li, G. M., and Huang, L. Therapeutic effect of Shengmai injection combined with routine treatment of chronic pulmonary heart disease. *Hebei. J. Tradit. Chin. Med.* 25, 32-33.

Li, D. S., and Gao, S. (2003). Clinical observation of Acanthopanax injection in the treatment of acute episodes of pulmonary heart disease. *Chin. J. Integr. Tradit. West. Med. Intensive. Crit. Care.* 10, 176.

Li, F. (2015). Clinical observation of 30 cases of chronic pulmonary heart disease treated by Shenmai injection combined with western medicine. *Yunnan. J. Tradit. Chin. Med. Mater. Med.* 36, 69-70.

Li, H. M., Zhang, H. R., and Tang, J. R. (2008). Clinical observation of 61 cases of Shenmai injection for pulmonary heart disease. *Shaanxi. J. Tradit. Chin. Med.* 29, 1572-1573.

Li, H. N. (2008). Clinical observation of 63 cases of Shenmai injection for pulmonary heart disease. *Intern. Med. J. Pract. Tradit. Chin. Med. Sc.* 22, 20.

Li, J. H., and Feng, Y. G. (2004). Clinical observation of 31 cases of Shengmai injection for pulmonary heart disease. *J. North. China. Coal. Med. Coll.* 6, 152-153.

Li, L. Z. (2007). Clinical observation of 40 cases of Shenfu injection for pulmonary heart disease. *Henan. Tradit. Chin. Med.* 27, 73-74.

Li, S. Y., Gao, X. L., and Zhang, J. X. (2012). Clinical study of Shenmai injection in the treatment of chronic pulmonary heart disease. *J. Clin. Pulm. Med.* 17,1214-1215.

Li, X. (2004). Therapeutic effect of 38 cases of Shenqi Fuzheng injection for pulmonary heart disease. *Mod. Med. Health.* 20, 1264.

Li, X. M., and Zhai, T. J. (2006). Effect of Shenfu injection on the cardiopulmonary function in the acute stage of pulmonary heart disease. *Chongqing. Med. J.* 35, 1219-1221.

Li, Y. H., Liu, Q. R., and Wang, J. B. (2004). Clinical observation of 36 cases of Astragalus injection for acute exacerbation of pulmonary heart disease. *J. Emerg. Syndromes. Tradit. Chin. Med.* 13, 220-221.

Li, Y. Q. (2009). Therapeutic effect of 26 cases of Shengmai injection for pulmonary heart disease. *Chin. Med. Mod. Dis. Educ. China.* 7, 133.

Li, Y. Q. (2014). Study on the effect of integrated traditional Chinese and western medicine in patients with pulmonary heart disease. *Cardiovasc. Dis. J. Integr. Tradit. Chin. West. Med.* 2, 63-64.

Lin, B., and Zeng, K. X. (2017). Clinical observation of 30 patients with pulmonary heart disease decompensation treated with Shenfu injection. *Yunnan. J. Tradit. Chin. Med. Mater. Med.* 38, 37-38.

Lin, H. Q., Guo, Z. J., Wu, Y. T., and Wu, Y. S. (2014). Effect of Dazhuhongjingtian injection on the right cardiac function in elderly patients with pulmonary heart disease. J. *Chin. Med. Mater.* 11, 2123-2124.

Liu, B. H. (2012). Clinical observation of Shenmai injection for severe pulmonary heart disease and heart failure. *Med. Front.* 2, 230-231.

Liu, H. L., and Lan, J. (2010). Clinical observation of 60 cases of Shengmai injection in the treatment of acute episodes of pulmonary heart disease. *Med. Inf.* 23, 111.

Liu, M. S. (2012). Therapeutic effect of Astragalus injection on chronic pulmonary heart disease. *Qinghai. Med. J.* 42, 90-91.

Liu, Z. Q. (2004). Observation on curative effects of Shengmai injection in treating acutely deteriorative phase of chronic pulmonary heart disease. *Prog. Pharm. Sci.* 28, 179-181.

Lu, Q., and Yang, L. (2014). Shenfu injection on hemorrheology in patients with acute corpulmonale. *World. Chin. Med.* 9, 1310-1313.

Lu, Y. H., and Wu, D. Y. (2001). Observation on chronic pulmonary heart disease and hemorheology of Shenmai injection. *J. Zhejiang. Tradit. Chin. Med. Coll.* 25, 36-37.

Luo, W. H. (2010). Therapeutic effect of Shenqi Fuzheng injection for pulmonary heart disease. *Qinghai. Med.* J. 40, 72.

Lv, G. M., and Jin, M. Y. (2011). Clinical observation of 50 cases of Shenmai injection on acute episode of chronic pulmonary heart disease. *Chin. Med. Mod. Dis. Educ. China.* 9, 57.

Ma, C. H. (2014). Clinical observation of Shenmai injection on acute episode of chronic pulmonary heart disease. *Neimonggu. J. Tradit. Chin. Med.* 32, 24.

Ma, D. T., Wang, F., Yu, P., Li, M., and Cheng, W. B. (2006). Therapeutic effect of Shengmai injection on chronic pulmonary heart disease with heart failure. *Shandong. Med. J.* 46, 28-29.

Ma, L., Yu, J. L., and Han, Y. F. (2005). Clinical observation of Astragalus injection in the treatment of pulmonary heart disease with heart failure. *Henan. Tradit. Chin. Med.* 25, 78-79.

Ma, Z. P., Cheng, X. H., and Wang, L. (2001). Clinical observation of 61 cases of chronic pulmonary heart disease with Huangqi injection. *Shaanxi. J. Tradit. Chin. Med.* 22, 279-280.

Mei, Y., and Li, Z. G. (2014). Clinical observation of 50 cases of Shenmai injection for pulmonary heart disease. *Med. Hum.* 27, 215.

Ni, X. Z. (2013). Effect of Shenmai injection on patients with chronic pulmonary heart disease. *Strait. Pharm. J.* 25, 200-201.

Qian, X. L. (2015). Clinical efficacy of Shenfu injection in the treatment of chronic pulmonary heart disease combined with heart failure. *For. All. Health.* 9, 112-113.

Qiu, Y. L., and Qiu, L. L. (1996). Observation on the effect of Ciwujia injection on 27 cases of pulmonary heart disease. *Clin. Focus.* 11, 800.

Ru, H. G. (2011). Effect of Shenfu injection on pulmonary heart disease with heart failure. *Zhejiang. J. Tradit. Chin. Med.* 37, 134.

Shen, X. X., Zu, J., Chen, M., Wei, Y., and Sun, Y. Y. (2011). Clinical observation of Shenfu in the treatment of chronic pulmonary heart disease. *J. Clin. Pulm. Med.* 16, 1686-1687.

Shi, B. (2003). Therapeutic effect of Shenmai injection on 42 patients with pulmonary heart failure. *Mod. J. Integr. Tradit. Chin. West. Med.* 12, 2179.

Shi, J. P., Liu, P. Z., and Kong, Y. H. (2017). Clinical observation of Shenmai injection for pulmonary heart disease. *Chin. Med. J. Metall. Ind.* 34, 148.

Song, B. (2011). Chronic lung source heart disease 48 example clinical analyses. *Chin. Manipulation. Qi. Gong. Ther.* 7, 43-44.

Song, Z. B. (1999). Therapeutic effect of Shenmai injection on 37 patients with chronic pulmonary heart failure. *Chin. J. Integr. Tradit. West. Medicine. Intensive. Crit. Care.* 6, 283.

Sun, G. Y., and Sun, L. (2002). Treatment of 40 cases of chronic pulmonary heart disease by Astragalus injection. *Med. J. Liaoning.* 16, 223.

Sun, Q., Zhang, B. L., and Sun, L. Q. (1998). Clinical observation of pulmonary heart disease treated with Huangqi injection. *J. Emerg. Syndromes. Tradit. Chin. Med.* 7, 206.

Sun, T. Y., and Zhou, X. L. (2003). Clinical observation of Shenmai injection on acute episode of chronic pulmonary heart disease. *Anhui. Med. Pharm. J.* 7, 187-188.

Sun, T., and Sun, J. (2009). Therapeutic effect of Shengmai injection on chronic pulmonary heart disease with heart failure. *J. Emerg. Syndromes. Tradit. Chin. Med.* 18, 1987-1988.

Wan, Q., Zhang, Q., and Zheng, Q. W. (2009). Observation on the curative effect of Shenmai injection on acute attack of chronic pulmonary heart disease. *New. Chin. Med.* 40, 671-672.

Wang, B., Chen, K. L., Deng, S. Z., Geng, L. M., and Tang, H. (1999). Clinical observation of 51 cases of pulmonary heart disease combined Huangqi injection with western medicine. *J. Pract. Tradit. Chin. Med.* 15, 21-22.

Wang, H. (2011). Therapeutic effect of Shenmai injection on patients with chronic cor pulmonale. *China. Trop. Med.* 11, 1404-1405.

Wang, K. X. (2000). Clinical observation of 40 cases of pulmonary heart disease combined with Chinese and western medicine. *Mod. J. Integr. Tradit. Chin. West. Med.* 9,1337.

Wang, L. (2007). Effect of Astragalus injection on heart failure serum enzyme caused by pulmonary heart disease. Intern. *Med. J. Pract. Tradit. Chin. Med. Sci.* 21, 79-80.

Wang, L. M. (2012). Clinical observation of 42 cases of Shenmai injection for pulmonary heart disease. *China. Health. Nutr.* 21, 490.

Wang, M. (2004). Clinical observation of Shenmai in treatment of pulmonary

Wang, S. H., and Liu, D. X. (2008). Effects of Shenmai injection on left cardiac function in elderly patients with chronic pulmonary heart disease. *Guide. China. Med.* 6, 106.

Wang, W., and Chen, K. (2004). Effects of Astragalus injection on endothelin -1 caused by pulmonary heart disease. *Chin. J. Integr. Med. Cardio.* 2, 567.

Wang, X. (2008). Clinical analysis of 70 cases of pulmonary heart disease treated by Chinese and western medicine. *J. Clin. Exp. Med.* 7, 167.

Wang, X. M. (2016). Effects of Shenmai injection on the treatment of pulmonary heart disease and its effects on heart and lung function. [dissertation]. [China]: Chengdu College of Traditional Chinese Medicine.

Wu, D. J., and Deng, Y. Y. (2011). Clinical observation of 32 cases of Shenfu injection for pulmonary heart disease with heart failure. *J. Emerg. Syndromes. Tradit. Chin. Med.* 20, 2022-2023.

Wu, L. W. (2004). Observation on the curative effect of Huangqi injection with pulmonary heart disease. *Xinjiang. Med. J.* 34, 107-108.

Wu, Q. (2003). Therapeutic effect of Shenmai injection on chronic pulmonary heart disease with heart failure. *Henan. Tradit. Chin. Med.* 23, 65-66.

Xiao, G. Z. (2009). Observation on the curative effect of Shenmai injection on heart failure of pulmonary heart disease. *J. Liaoning. Univ. Tradit. Chin. Med.* 11, 123-124.

Xiao, W., Lei, R., Huang, X. X., Chen, Z. P., Xu, J. X., and Ma, J. L. (2012). Effect of Astragalus injection on acute exacerbation of chronic pulmonary heart disease. *Mod. J. Integr. Tradit. Chin. West. Med.* 21, 1197-1198.

Xie, Y. B., Li, H. D., and Li, P. (2009). Clinical observation of Shenmai injection on acute episode of chronic pulmonary heart disease. *Lingnan. J. Emerg. Med.* 14, 307-308.

Xu, L. N. (2011). Clinical observation of Shenmai injection in treating 46 cases of hronic cor pulmonale with heart failure. *Chin. J. Mod. Drug. Appl.* 5, 80-81.

Xue, Z. F., and Tang, Y. Q. (2005). Correlation of Shenmai injection on hemorheology and blood gas analysis of pulmonary heart disease. *Chin. J. Hemorheol.* 15, 258-259.

Yang, J., and Lan, D. (2017). Experimental observation on the influence of Shenmai Injection on blood flow function in patients with chronic pulmonary heart disease. *China. Mod. Med.* 24, 109-111.

Yang, Z. Y. (1997). Observation on the effect of Astragalus injection on 65 cases of pulmonary heart disease. *Liaoning. J. Tradit. Chin. Med.* 24, 261.

Ye, P. X. (1999). Therapeutic effect of Shenmai injection on chronic pulmonary heart disease with heart failure. *Cent. Plains. Med.* J. 27, 6-7.

Ye, P. X., and Luo, B. C. (1999). Effect of Shenmai injection on hemorheology of pulmonary heart disease. *Zhejiang. Pract. Med.* 4, 35-36.

Ye, Y. L., and Zhang, W. F. (2012). Effect of Shenmai injection on the expression of pac-1 and CD62p in patients with acute exacerbation of pulmonary heart disease. *Chin. Mod. Doctor.* 50, 56-58.

Yin, F. J. (2011). Clinical observation of Shenmai injection on acute episode of chronic pulmonary heart disease. *Med. Inf.* 24, 212-213.

Yin, J., and Wei, Q. (2002). Therapeutic effect of Astragalus membranaceus injection on heartfailure caused by pulmonary heart disease. *Hebei. J. Tradit. Chin. Med.* 24, 308.

Yu, J. X. (2005). Therapeutic effect of Shengmai injection on chronic pulmonary heart disease with hyperviscosity. *Guangxi. Med. J.* 27, 1606-1607.

Zhang, D. J. (2002). Observation on the efficacy of Huangqi injection in treating 42 cases of pulmonary heart disease. *Mod. J. Integr. Tradit. Chin. West. Med.* 11, 408.

Zhang, L. J., Wang, G. X, and Zhang, G. S. (2000). Observation on the curative effect of Huangqi injection with pulmonary heart disease. *Tibetan. J. Med.* 21, 14-15.

Zhao, L. Y., and Kang, W. (2000). Observation on the effect of Huangqi injection on 16 cases of pulmonary heart disease. *Mod. J. Integr. Tradit. Chin. West. Med.* 9,1274-1275.

Zhao, Y. H. (2011). Observation of Shenmai injection in treatment of chronic pulmonary heart disease*. Chin. Mod. Doctor.* 49, 132-133.

Zhen, Z. X., and Zhang, W. F. (2013). Clinical observation of Shenmai injection in treatment of chronic pulmonary heart disease complicated with right ventricular failure. *Chin. J. Mod. Drug. Appl.* 7, 38-39.

Zheng, Q. W., and Huang, B. (2006). Clinical observation on the acute onset stage of pulmonary heart disease with Shenmai injection. *J. Guiyang. Coll. Tradit. Chin. Med.* 28, 30-31.

Zheng, W. T., and Qian, W. Y. (2005). Effect of Shenmai injection on serum SOD and MDA content in patients with pulmonary heart disease*. J. Binzhou. Med. Coll.* 28, 208-209.

Zhou, B., Zhang, X. Y., Chang, Z. F., Zhang, J. E., and Tang, Z. Y. (1999). Clinical observation of 64 cases of dilated cardiomyopathy treated with Astragalus injection. *Chin. J. New. Drugs. Clin. Rem.* 18, 374-375.

Zhou, L. J., Wang, Y. C., and Zhou, S. X. (2003). A summary of 60 cases of chronic pulmonary heart disease treated by Astragalus injection. *Hunan. J. Tradit. Chin. Med.* 19, 3.

Zhu, J. F. (2015). Clinical observation of 76 cases of Shenmai injection for pulmonary heart disease. Zhejiang Severe Medicine Academic Annual Conference.

Zhu, W., and Wu, D. J. (2001). Observation on chronic pulmonary heart disease and hemorheology of Astragalus injection. *Mod. J. Integr. Tradit. Chin. West. Med.* 10, 2131-2132.

Zhu, X. F. (2001). Observation on the therapeutic effect of Shenmai injection in the treatment of chronic pulmonary heart disease. *Chin. Gen. Pract.* 4, 751.

Zhu, Y. H., Cai, W. R., He, Y. S., and Xu, J. (1999). Observation on curative effects of Acanthopanax root injection on treating acutely deteriorative phase of cor pulmonale. *Chin. J. Integr. Tradit. West. Med. Intensive. Crit. Care.* 6, 502-504.

Zhu, Y. H., Cai, W. R., Wang, Z., and Zhu, Y. H. (2005). Effect of Astragalus injection on cardiopulmonary function of pulmonary heart disease. *J. Zhejiang. Tradit. Chin. Med. Coll.* 29, 20-21.

Zhu, Z. Y. (2001). Clinical observation of Astragalus injection for acute exacerbation of pulmonary heart disease. *Chin. J. Integr. Med.* 21, 268.

Zou, Q. (2002). Clinical observation of 40 cases of chronic pulmonary heart disease with Huangqi injection. *Fujian. J. Tradit. Chin. Med.* 33, 29-30.


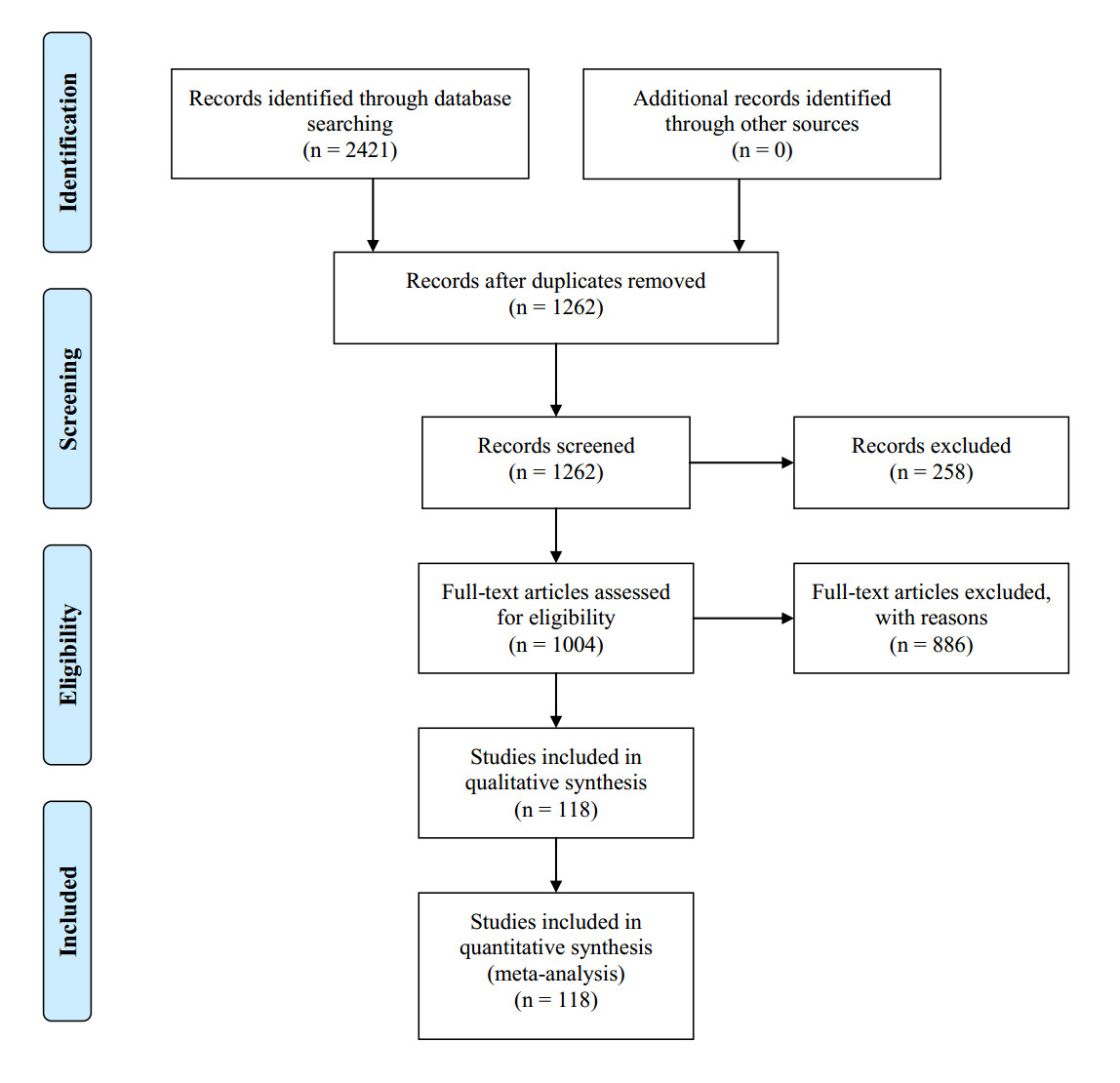


**Supplementary Figure 1.** Flow chart of the search for included randomized controlled trials.


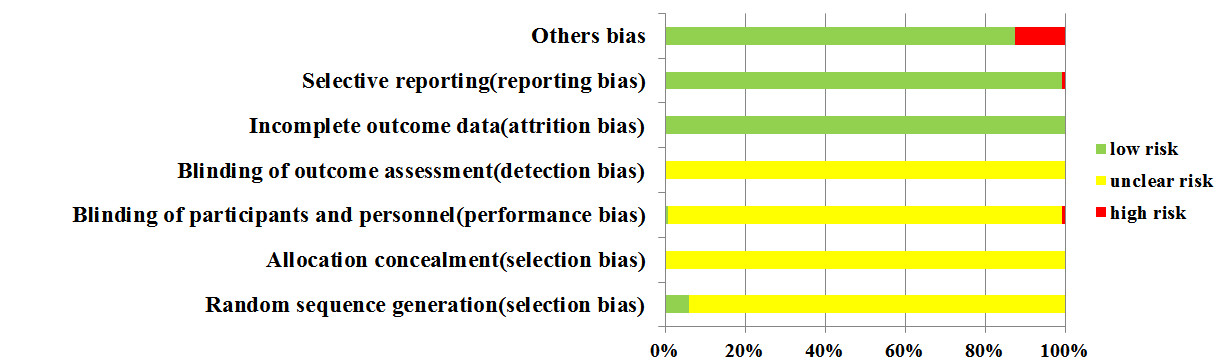


**Supplementary Figure 2.** Risk of bias graph.
